# Multicenter study on magnesium isoglycyrrhizinate preventing novel antitumor-induced liver injury in hematological malignancies

**DOI:** 10.3389/fmed.2026.1759611

**Published:** 2026-06-26

**Authors:** Chen Liang, Li Liu, Rongli Zhang, Donglin Yang, Yi He, Aiming Pang, Sizhou Feng, Mingzhe Han, Erlie Jiang

**Affiliations:** 1 State Key Laboratory of Experimental Hematology, National Clinical Research Center for Blood Diseases, Haihe Laboratory of Cell Ecosystem, Institute of Hematology & Blood Diseases Hospital, Chinese Academy of Medical Sciences & Peking Union Medical College, Tianjin, China; 2 Tianjin Institutes of Health Science, Tianjin, China

**Keywords:** drug-induced liver injury, hematological malignancies, hepatotoxicity, magnesium isoglycyrrhizinate, retrospective analysis

## Abstract

**Background:**

Antineoplastic agents significantly contribute to drug-induced liver injury (DILI). This study evaluates magnesium isoglycyrrhizinate (MgIG) for preventing DILI in hematological malignancy patients receiving novel antitumor therapies.

**Methods:**

This multicenter retrospective analysis included hematological malignancy patients treated with hepatoprotective agents across 13 Chinese centers (December 2023–February 2024). Primary outcomes assessed liver injury incidence and severity at 21, 30, and 60 days; secondary outcomes evaluated safety.

**Results:**

Propensity score-matched cohorts (MgIG = 324, control = 182) showed balanced baselines. MgIG recipients had higher chemotherapy (91.4 vs. 64.3%, *P* < 0.001) and Venetoclax exposure (26.2 vs. 2.2%, *P* < 0.001). Baseline AE incidence was higher with MgIG (4.6 vs. 0.5%, *P* = 0.014), but subsequent follow-ups showed comparable AE rates. Hepatic injury incidence was similar (4.2 vs. 4.0%, *P* > 0.05). Controls exhibited greater ALT, AST and γ-GGT elevations at day 30 and γ-GGT elevation at day 60 (all *P* < 0.05). MgIG reduced overall hepatic AEs (48.0 vs. 66.7%, *P* < 0.001), driven by fewer grade 1–2 abnormalities (43.5 vs. 60.3%, *P* < 0.001), though grade ≥2 events remained comparable. Interim assessments revealed higher hepatic AE rates in controls at day 21 (54.5 vs. 37.1%) and day 30 (55.7 vs. 34.1%; *P* < 0.001), with elevated grade ≥2 AEs by day 60 (18.6 vs. 8.6%, *P* = 0.022).

**Conclusions:**

Prophylactic MgIG attenuates antineoplastic therapy-induced ALT/AST/TBiL elevations in hematological malignancies, demonstrating hepatoprotective efficacy. While its impact on overall DILI incidence remains unclear, longitudinal data suggest clinically meaningful mitigation of hepatotoxicity severity in high-risk subgroups during critical phases.

## Introduction

Herapeutic drugs and targeted therapies, encompassing both small-molecule and large-molecule agents, as well as immune checkpoint inhibitors (ICIs), have the potential to induce liver injury. In clinical trials, the reported incidence of all-grade liver injury varies widely among different small-molecule targeted therapies, such as tyrosine kinase inhibitors (TKIs), ranging from 5 to 55% (1–6). The incidence of ICI-associated hepatitis is contingent upon factors such as the type of ICI, dosage, and whether administered as monotherapy or in combination therapy (7, 8).

There is currently limited overall evidence on whether the preventive use of drugs for DILI associated with novel antitumor agents in patients with hematological disorders. Relevant guidelines and consensuses suggest considering preventive use of such drugs in special clinical scenarios, such as for high-risk individuals or those receiving ultra-high doses of antitumor therapy (9). For haemato-oncology patients who experience DILI, especially severe liver injury, during their initial antitumor treatment and cannot adjust their treatment plan subsequently, re-exposure to the same antitumor regimen (rechallenge) must be carefully considered due to the potential risk of even more severe liver injury.

Among the extensive array of drugs used to treat liver injury, only a limited number have been investigated for their potential in preventing DILI. Research on magnesium isoglycyrrhizinate (MgIG) for the prevention and treatment of liver injury in acute leukemia patients undergoing high-dose chemotherapy indicated its effectiveness in mitigating adverse reactions associated with chemotherapy for hematological malignancies, enhancing therapeutic efficacy while reducing toxicity (10). Prophylactic administration of MgIG has been shown to reduce the incidence of DILI during neoadjuvant chemotherapy for breast cancer, particularly in patients with abnormal hepatitis B markers (11). The most recent meta-analysis found that preventive use of MgIG outperforms other liver-protecting drugs in reducing the incidence of chemotherapy-induced liver dysfunction and improving levels of alanine aminotransferase (ALT), alkaline phosphatase (ALP), and total bilirubin (TBIL) in cancer patients, without increasing adverse reactions (12). While some studies have initially shown the efficacy and safety of prophylactic hepatoprotective effect of MgIG, the overall evidence is still scarce. This retrospective study, focusing on patients with hematological malignancies, investigated the effectiveness and safety of MgIG in preventing liver injury related to novel antitumor agents in this cohort, aiming to offer more evidence-based insights for its clinical use in liver protection.

## Materials and methods

### Study subjects

This multicenter, retrospective analysis was conducted in 13 medical centers in China. Patients with malignant hematological disorders were consecutively enrolled between December 2023 and February 2024. Patients must meet all of the following criteria to be enrolled in this study: 1) diagnosis of malignant hematologic disease (including but not limited to lymphoma, myeloma, leukemia); 2) age ≥18 years; 3) anticancer treatment regimen including at least one novel anticancer drug (Supplementary Table 1); 4) one of baseline levels of TBiL, ALT, aspartate aminotransferase (AST) or ALP ≤ 1.0 × upper limit of normal (ULN) prior to hepatoprotective agents administration. Subjects who meet any of the following criteria shall be excluded from participation in this study: 1) patients receiving hepatoprotective agents other than MgIG (e.g., polyene phosphatidylcholine, reduced glutathione, tiopronin, etc.); 2) those undergoing concurrent hepatic radiotherapy; 3) patients receiving concurrent cellular immunotherapy; 4) patients with active hepatitis B virus (HBV) or hepatitis C virus (HCV) replication requiring antiviral therapy and 5) those lacking post-treatment safety follow-up records. The patients with hematological malignancies who commenced MgIG administration for hepatoprotection on or prior to the initial day of anticancer treatment constituted the observation group (MgIG group), whereas those who did not undergo any hepatoprotective therapy were assigned to the control group.

This study was conducted in strict accordance with the principles outlined in the Declaration of Helsinki (1996 edition), the Good Clinical Practice (GCP) guidelines issued by the State Food and Drug Administration (SFDA), and all relevant regulatory frameworks. The study has received approval from the lead institutions, the Chinese Academy of Medical Sciences Hospital of Hematology (approval number QTJC2024027-EC-1), as well as the ethics committees of each participating center. Stringent measures were implemented to ensure the confidentiality and privacy of patient data to protect personal identifying information. In consideration of the retrospective nature of this study, the requirement for obtaining informed consent from participants was dispensed with.

### Data collection

A comprehensive collection of patients' general clinical data was undertaken, encompassing demographic details, past medical history, current medical status, prevailing anticancer treatment protocols, laboratory indices, and adverse events. Additionally, vigilant monitoring was implemented to detect any drug-related adverse reactions occurring during the anticancer therapeutic process.

### Outcomes and definitions

The primary outcome was to evaluate the incidence and severity of liver injury occurring within 21, 30, and 60 days of preventive treatment with MgIG, utilizing the National Cancer Institute Common Terminology Criteria for Adverse Events (NCI-CTCAE) version 5.0 as the assessment standard for liver injury. The secondary outcome focused on safety evaluations, encompassing the documentation of adverse events (AEs) with respect to their type, incidence, severity, duration, and association with the investigational drug. Liver injury was defined based on the fulfillment of any one of the following criteria: (1) ALT ≥ 5 × ULN; (2) ALP ≥ 2 × ULN, particularly when accompanied by an increase in gamma-glutamyltransferase (γ-GGT) and excluding elevations attributable to bone diseases; or (3) ALT ≥ 3 × ULN concurrently with TBil ≥ 2 × ULN. The presence of any one of these criteria was sufficient for a diagnosis of acute drug-induced liver injury (13).

### Data analysis

Statistical analyses were performed using SPSS software (version 26.0). Quantitative variables conforming to a normal distribution were described using the mean ± standard deviation (SD) and compared using the *t*-test. For quantitative variables with a skewed distribution, the median and interquartile range (IQR) were employed for description, with comparisons made using the Wilcoxon rank-sum test. Categorical variables were presented as frequencies and percentages, with comparisons conducted using Fisher's exact test. Propensity Score Matching (PSM) was utilized to equilibrate the baseline characteristics between the MgIG and control groups. The variables integrated into the propensity score (PS) model comprised gender, age, body mass index (BMI), receipt of immunotherapy, number of targeted therapeutic agents administered, presence of fatty liver disease, diabetes mellitus, hypertension, and hyperlipidemia. The nearest neighbor matching algorithm was applied, with a matching ratio of 2:1 between the groups, and a caliper value set at 0.2 standard deviations of the propensity score to ensure adequate matching. A standardized mean difference (SMD) of less than 0.1 was regarded as indicative of a good balance between the groups. Statistical significance was established at a *P*-value less than 0.05.

## Results

### Demographic and clinical characteristics at baseline

**Figure 1:**
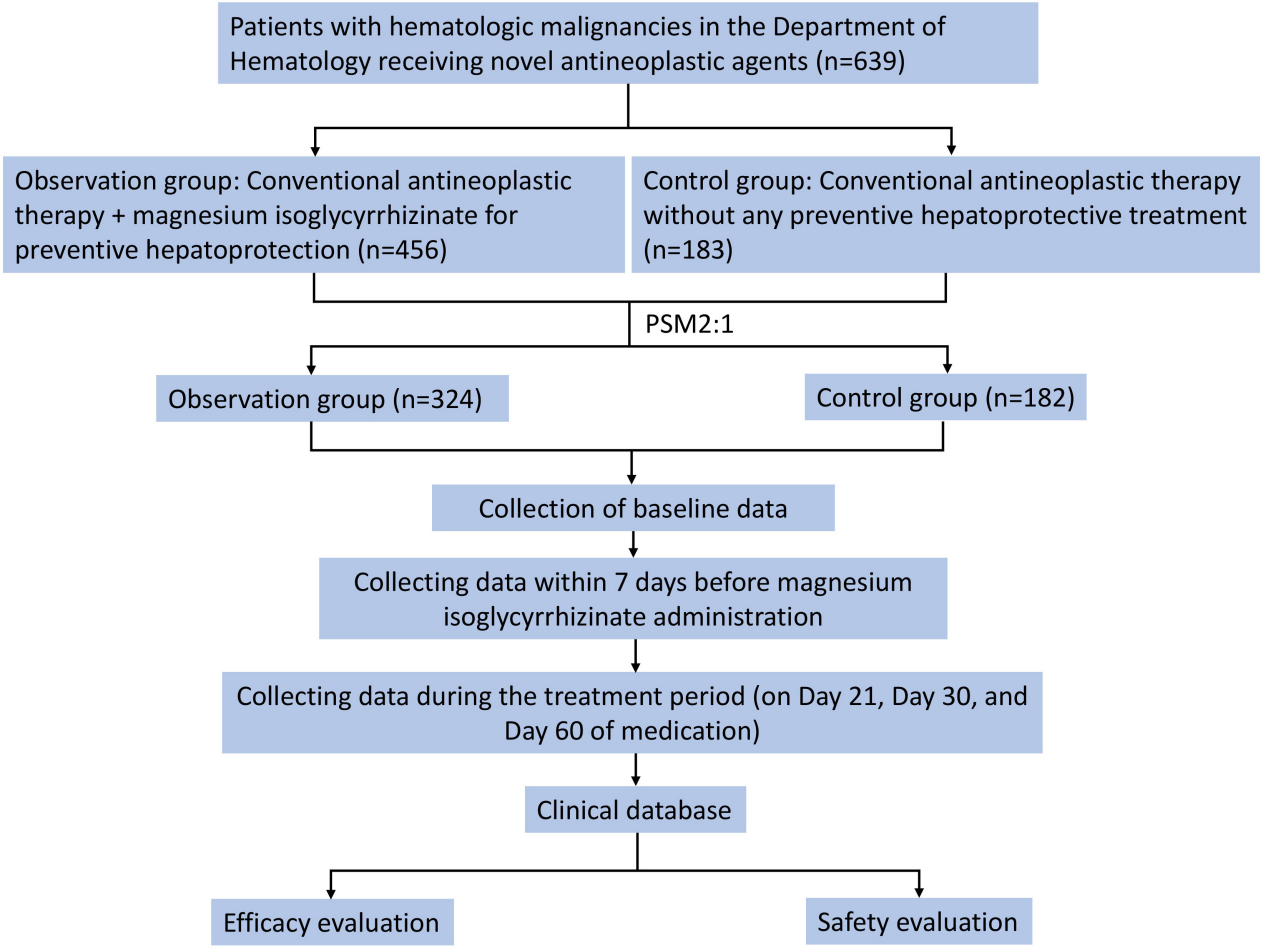
Study flow and patient selection process. Flowchart illustrating enrollment, group allocation, data collection, and propensity score matching (PSM) of patients with hematological malignancies receiving novel antineoplastic agents. A total of 639 patients were initially enrolled; after 2:1 PSM, 324 patients in the MgIG group and 182 patients in the control group were included in the final analysis.

A total of 639 patients with hematological malignancies receiving novel antineoplastic agents were enrolled. Patients were allocated into two groups: the observation group received conventional antineoplastic therapy plus magnesium isoglycyrrhizinate for preventive hepatoprotection, while the control group received only conventional antineoplastic therapy without any preventive hepatoprotection. Baseline data were collected within 7 days before MgIG administration, and clinical data during treatment were recorded at Day 21, Day 30, and Day 60. All efficacy and safety evaluations were conducted after propensity score matching (PSM) at a ratio of 2:1, yielding a final matched cohort of 324 patients in the MgIG group and 182 patients in the control group (Figure 1).

Among the 639 included patients, 456 received MgIG for hepatoprotection on or before the first day of anticancer treatment, and 183 did not receive any hepatoprotective therapy. Preliminary statistical analysis identified significant baseline discrepancies (*P* < 0.05) in gender, anthropometrics (weight/BMI), and clinical profiles (hyperlipidemia, surgical/chemotherapy history) between the two groups (data not shown). These covariate imbalances require statistical matching to ensure valid intergroup comparisons. PSM was utilized to balance baseline characteristics between the MgIG group and the control group. The variables in the Propensity Score (PS) model encompassed gender, age, BMI, immunotherapy status, number of targeted agents, liver diseases, diabetes, hypertension, and hyperlipidemia. Employing nearest-neighbor algorithm with a ratio of 2:1 and a caliper of 0.2 times of standard deviation (SD) of the propensity score, optimal balance was achieved for MgIG (*n* = 324) and control group (*n* = 182) with all standard mean difference (SMD) < 0.1 (Supplementary Figure 1).

The baseline characteristics, including gender, age, height, weight, BMI, type of hematological malignancy, and medical histories (liver disease, diabetes, hyperlipidemia, allergies, surgical history, and drug allergies), were comparable between the two matched groups, showing no statistically significant differences (*P* > 0.05). Neither group received immunotherapy, and only a scant number of patients underwent radiotherapy (0.6 vs. 1.1%, *P* > 0.05). Remarkably, the MgIG cohort demonstrated a significantly higher proportion of patients receiving chemotherapy compared to the control group (91.4 vs. 64.3%, *P* < 0.001), along with a notably elevated percentage of patients with a prior history of chemotherapy (5.9 vs. 0.0%, *P* < 0.001). With regard to targeted therapy and the utilization of targeted drugs, no statistically significant difference was observed between the two groups. Nonetheless, a significantly greater proportion of patients in the MgIG group were administered Venetoclax (26.2 vs. 2.2%, *P* < 0.001), whereas the control group exhibited a markedly higher percentage of patients treated with Ocalivumab (0.0 vs. 11.0%, *P* < 0.001; Table 1).

**Table 1:** Baseline characteristics of included patients.

Characteristics	MgIG (*N* = 324)	Non-MgIG (*N* = 182)	*p*-value
Gender, *n* (%)			
Male	187 (57.70)	113 (62.10)	0.347^1^
Female	137 (42.30)	69 (37.90)	
Mean age (SD), years	57.63 (15.01)	58.30 (17.25)	0.660^2^
Mean height (SD), cm	165.85 (8.70)	165.90 (8.66)	0.955^2^
Mean weight (SD), kg	64.19 (10.72)	63.10 (10.00)	0.252^2^
BMI, mean (SD)	23.28 (3.04)	22.92 (3.15)	0.202^2^
Hematological malignancy types, *n* (%)			
Lymphoma	110 (34.00)	71 (39.00)	0.503^1^
Multiple myeloma	93 (28.70)	46 (25.30)	
Leukemia	121 (37.30)	65 (35.70)	
Hepatitis B, *n* (%)	22 (6.80)	7 (3.80)	0.232^1^
Other liver diseases, *n* (%)	170 (52.50)	69 (37.90)	0.002^1^
Diabetesmellitus, *n* (%)	24 (7.40)	18 (9.90)	0.401^1^
Hypertension, *n* (%)	54 (16.70)	38 (20.90)	0.280^1^
Hyperlipidemian (%)	1 (0.30)	1 (0.50)	>0.999^1^
History of allergies, *n* (%)	3 (0.90)	1 (0.50)	>0.999^1^
History of surgery, *n* (%)	48 (14.80)	12 (6.60)	0.006^1^
History of chemotherapy, *n* (%)	19 (5.90)	0 (0.00)	< 0.001^1^
History of drug allergies, *n* (%)	11 (3.40)	3 (1.60)	0.397^1^
Missing	1	0	
Anti-tumor treatment, *n* (%)			
Chemotherapy	296 (91.40)	117 (64.30)	< 0.001^1^
Immunotherapy	0 (0.00)	0 (0.00)	>0.999^1^
Radiotherapy	2 (0.60)	2 (1.10)	0.621^1^
Targeted cancer therapy	324 (100.00)	181 (99.50)	0.360^1^
Number of targeted therapeutic agents			
0	0 (0.00)	1 (0.50)	0.720^1^
1	288 (88.90)	159 (87.40)	
2	35 (10.80)	23 (12.60)	
3	1 (0.30)	0 (0.00)	
Name of targeted therapeutic agents			
Rituximab	96 (29.60)	46 (25.30)	0.305^1^
Venetoclax	85 (26.20)	4 (2.20)	< 0.001^1^
Bortezomib	43 (13.30)	20 (11.00)	0.486^1^
Lenalidomide	27 (8.30)	25 (13.70)	0.067
Daratumumab	16 (4.90)	15 (8.20)	0.175^1^
Pomalidomide	24 (7.40)	44 (2.20)	0.014^1^
Ruxolitinib	6 (1.90)	14 (7.70)	0.003^1^
Ocalivumab	0 (0.00)	20 (11.00)	< 0.001^1^
Obinutuzumab	11 (3.40)	5 (2.70)	0.796^1^
Flumatinib	5 (1.50)	8 (4.40)	0.076^1^
Dasatinib	7 (2.20)	3 (1.60)	>0.999^1^
Zanubrutinib	2 (0.60)	9 (4.90)	0.002^1^
Olabarrutinib	2 (0.60)	8 (4.40)	0.005^1^
Gilteritinib	4 (1.20)	3 (1.60)	0.706^1^
Imatinib	6 (1.90)	5 (2.70)	0.535^1^
Carfilzomib	5 (1.50)	0 (0.00)	0.165^1^
Blinatumomab	5 (1.50)	1 (0.50)	0.427^1^
Thalidomide	3 (0.90)	3 (1.60)	0.672^1^
Chidamide	4 (1.20)	3 (1.60)	0.706^1^
Ixazomib	3 (0.90)	0 (0.00)	0.556^1^
Selinexor	5 (1.50)	0 (0.00)	0.165^1^
Brentuximab vedotin	1 (0.30)	2 (1.10)	0.295^1^
Nilotinib	1 (0.30)	5 (2.70)	0.025^1^
Ibrutinib	0 (0.00)	2 (1.10)	0.129^1^

Data are presented as *n* (%) for category variables and mean (SD) for continuous variables.

Percentages and means are based on non-missing value.

^1^Fisher's exact test

^2^Welch two sample t-test

MgIG: magnesium isoglycyrrhizinate

### Hepatic injuries related to liver function indicators

**Figure 2:**
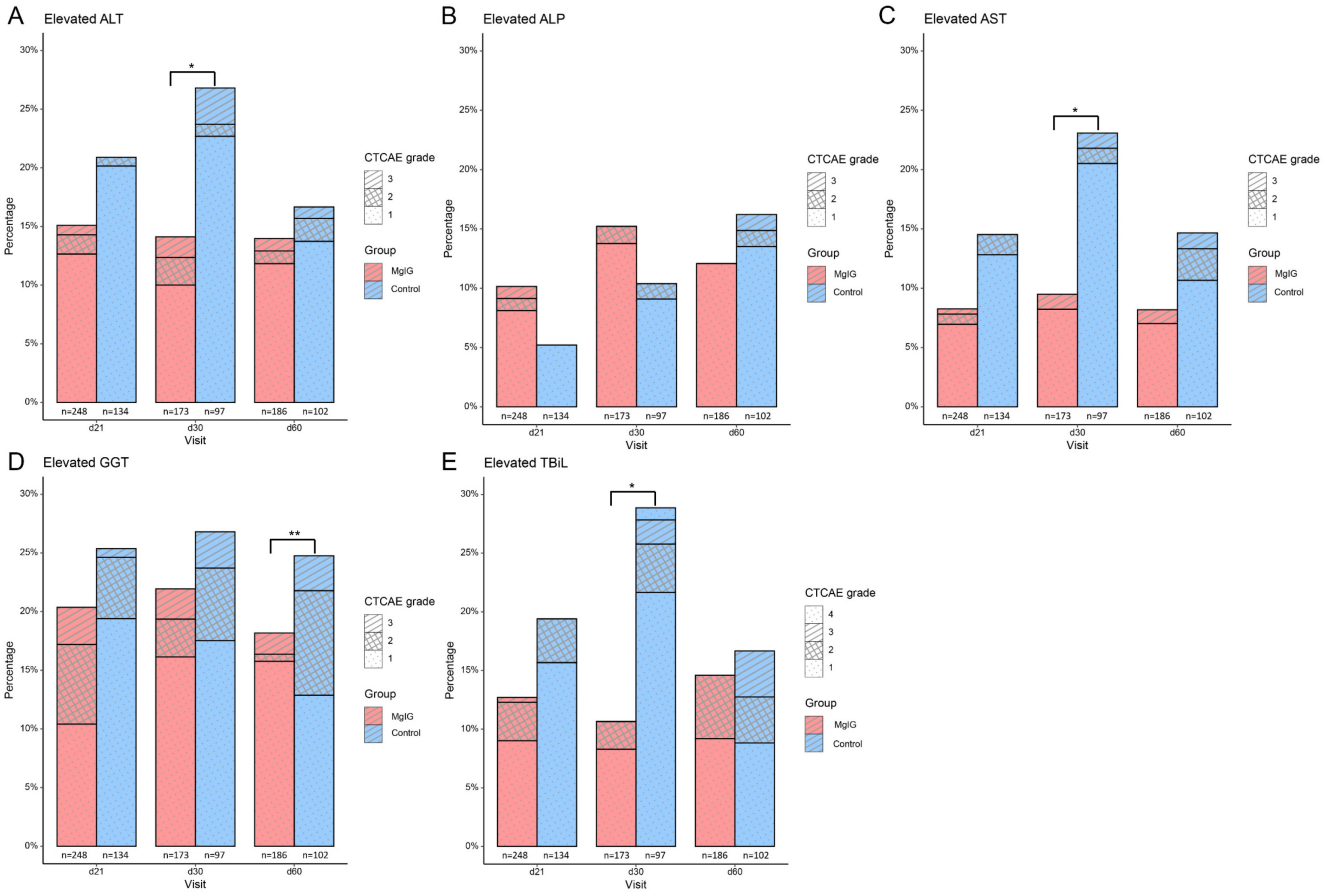
Intergroup comparison of **(A)** ALT; **(B)** ALP; **(C)** AST; **(D)** γ-GGT and **(E)** TBiL based on the CTCAE grading criteria. Comparisons of **(A)** ALT, **(B)** ALP, **(C)** AST, **(D)** γ-GGT, and **(E)** TBil between the MgIG group and control group at Day 21, Day 30, and Day 60. Grading was performed according to NCI-CTCAE version 5.0. Data are presented as the percentage of patients with elevated liver function indices at each grade level. *P < 0.05 vs. control group, **P < 0.01 versus control group.

According to predefined criteria, 13 (4.2%) in the MgIG group and 7 (4.0%) in the control group had hepatic injuries, with no statistically significant difference (*P* > 0.05; Supplementary Table 2). A comparative analysis was further conducted to assess the proportions and magnitudes of increase in ALT, AST, ALP, TBil, and γ-GGT, categorized according to the Common Terminology Criteria for Adverse Events (CTCAE) ver. 5.0 grading system, between the two groups at 21, 30, and 60 days following the initiation of treatment (Figure 2). The results revealed no statistically significant disparities in the elevations of these liver function indicators between the groups at the 21-day (*P* > 0.05; Figures 2A–E, leftmost part). Nonetheless, at the 30-day follow-up, the control group exhibited significantly greater increases in ALT, AST, and TBiL levels compared to the MgIG group (*P* < 0.05; Figures 2A, C, E, middle part). Furthermore, at the 60-day assessment, the control group demonstrated a significantly higher incidence of γ-GGT, as compared to the MgIG group (*P* = 0.005; Figure 2D, rightmost part).

### Comprehensive assessment of hepatic injurie severity

A comprehensive comparison of hepatic injury severity between the control and MgIG groups was summarized in Supplementary Table 3. A statistically significant disparity was identified between the control and the MgIG group (66.7 vs. 48.0%, *P* < 0.001). Specifically, the occurrence of grade 1–2 hepatic AEs was markedly higher in the control cohort compared to the MgIG group (60.3 vs. 43.5%, *P* < 0.001), while no statistically significant difference was discerned in the incidence of grade 2 or higher hepatic injuries (*P* > 0.05).

**Figure 3:**
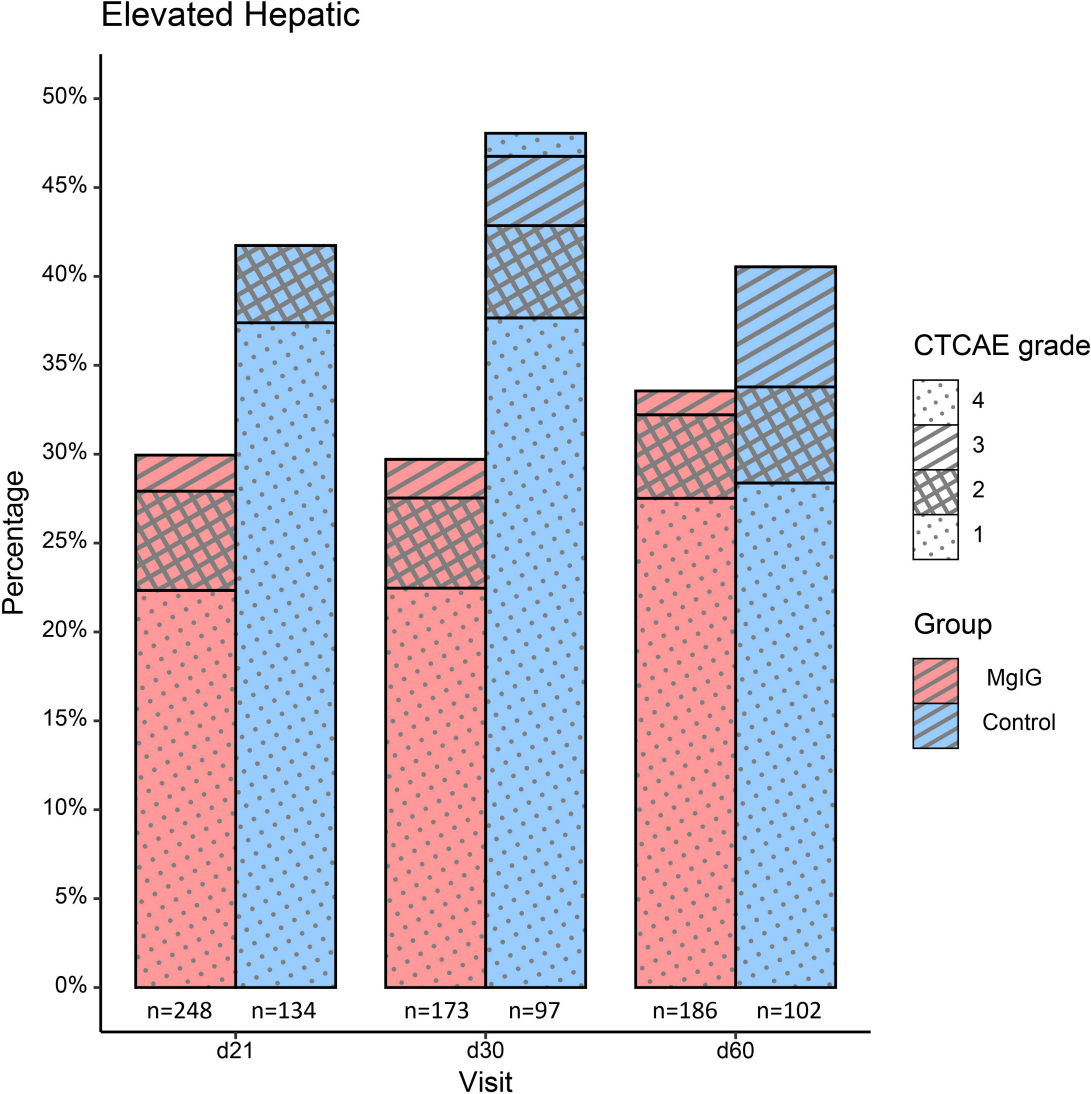
Temporal dynamics of hepatic adverse event severity in the MgIG and control groups. Incidence and severity distribution of liver function–related adverse events (AEs) at Day 21, Day 30, and Day 60. AEs were defined as any elevation in ALT, AST, ALP, TBil, or γ-GGT to grade ≥1 per CTCAE 5.0. The proportion of overall hepatic AEs, grade 1–2 AEs, and grade ≥2 AEs are shown at each time point.

Figure 3 further delineates the temporal dynamics of hepatic injury severity by evaluating AEs at day 20, 30, and 60. The analysis focuses on liver function parameters (ALT, AST, ALP, TBiL, and γ-GGT), with AEs defined as any parameter elevation to grade 1 or higher. The control cohort exhibited a significantly elevated incidence of hepatic injuries relative to the MgIG group at day 21 (54.5 vs. 37.1%, *P* < 0.001) and at day 30 (55.7 vs. 34.1%, *P* < 0.001). This significant disparity was particularly evident in the case of grade 1–2 liver function-related AEs, which were more prevalent in the control cohort (53.7 vs. 33.5%, *P* = 0.003 at day 21 and 48.5 vs. 31.2%, *P* < 0.001 at day 30). At the 60-day assessment, however, no statistically significant difference was observed in the overall incidence of hepatic AEs between the control and MgIG cohorts (47.1 vs. 40.9%, *P* > 0.05). Noteworthy, the control cohort demonstrated a significantly higher incidence of grade 2 or higher hepatic AEs compared to the MgIG group (18.6 vs. 8.6%, *P* = 0.022).

After propensity score matching, stratified analyses were performed by chemotherapy administration, hematologic cancer type and age subgroup according to the occurrence of liver function–related AE. In the chemotherapy-stratified subgroup analyses, the MgIG group with chemotherapy exhibited a significantly lower incidence of such AEs (*P* = 0.001). Differences in the worst grade and severity grouping were also statistically significant (*P* = 0.008 and *P* = 0.002, respectively). At Day 21, significant between-group differences were found in the occurrence of liver function–related AEs (*P* = 0.029). At Day 30, the incidence of liver function–related AEs and worst grade distribution differed significantly (*P* = 0.002 and *P* = 0.004, respectively), as did severity grade grouping (*P* = 0.002; Supplementary Table 4). Significant differences of the occurrence of liver function–related AE was only found in the MgIG and control group in leukemia (44.3 vs. 75.8%, *P* < 0.001), with significant differences also in the worst grade distribution (*P* < 0.001) and occurrence of AEs at Day 21 (*P* = 0.016), while no significant differences were found in ≥Grade 2 (*P* = 0.138) and ≥Grade 3 (*P* = 0.755) AEs for leukemia (Supplementary Table 5). In the age stratified analysis comparing participants aged < 60 and ≥60 years, significant between group differences were detected in the incidence of liver function related AEs across multiple time points. The control group consistently exhibited a higher incidence of such events, with rates of 70.5 vs. 52.4% in the MgIG group among patients < 60 years (*P* = 0.010) and 63.5 vs. 44.1% among those ≥60 years (*P* = 0.003). Significant differences were also observed in the distribution of the worst AE grade (*P* = 0.022 for < 60 years; *P* = 0.002 for ≥60 years), the incidence of AEs at day 21 (*P* = 0.018 for < 60 years; *P* = 0.037 for ≥60 years), the incidence of AEs at day 30 (*P* = 0.041 for < 60 years; *P* = 0.018 for ≥60 years), and the distribution of the worst event grade at day 30 (*P* = 0.030 for < 60 years; *P* = 0.008 for ≥60 years). A significant difference in the incidence of grade ≥2 liver function related AEs at day 60 was identified only in the ≥60 years subgroup (*P* = 0.020), whereas no such difference was observed in the < 60 years subgroup (*P* = 0.315; Supplementary Table 6). In further detailed subgroup analyses across age, chemotherapy status, and disease type, no statistically significant between-group differences were observed in overall liver function–related adverse events, worst grade distribution, severity stratification, or the incidence of grade ≥2 and grade ≥3 events at baseline and through Days 21, 30, and 60 (all *P* > 0.05), confirming that age, chemotherapy administration, and hematologic cancer type did not act as significant effect modifiers (Supplementary Tables 7–9). These findings support the consistency of the comparative liver safety profile of MgIG across all clinically relevant subgroups studied.

### Incidence of non-hepatic AE

The AEs observed during the study period were summarized in Supplementary Table 10. At baseline, a statistically significant higher incidence of AE was observed in the MgIG compared to the control group (*P* = 0.014). Nevertheless, no statistically significant differences in incidence of AE were evident between the two groups at the 21-day, 30-day and 60-day post-treatment follow-ups (*P* > 0.05). Additionally, no clinically meaningful disparities were observed in systemic manifestations, encompassing serum potassium concentrations, palpitations, fatigue, nausea, vomiting, diarrhea, pruritus, and rash, between the MgIG and the control groups.

MgIG, representing the fourth generation of glycyrrhizic acid formulations, contains 18α-glycyrrhizic acid as its sole active isomer, enabling safe and efficacious prevention and management of DILI through its hepatoprotective properties (14). In clinical practice, MgIG has consistently demonstrated favorable safety profiles, whether administered as monotherapy or in combination with other therapeutic strategies. A study investigating the efficacy and safety of MgIG in DILI treatment enrolled 82 patients, who were randomized into an observation group receiving MgIG alongside standard care and a control group receiving only standard care. The findings revealed that the incidence of adverse reactions in the MgIG-treated observation group was 7.32%, which was not statistically different from the 4.88% observed in the control group (*P* > 0.05). This underscores that MgIG does not elevate the risk of adverse reactions in DILI management (15). Our study also demonstrated no significant disparity in adverse reactions between the MgIG group and the control group among patients with malignant tumors.

## Discussion

Drug-induced liver injury (DILI) represents a formidable challenge in the realm of advanced cancer therapeutics. In recent years, immune checkpoint inhibitors (ICIs) and tyrosine kinase inhibitors (TKIs) have gained widespread application in the treatment of both solid tumors and hematological malignancies (16–19). Nonetheless, akin to conventional chemotherapeutic drugs, these targeted therapeutic modalities have also been implicated in the induction of DILI (20–23). As such, the prevention of DILI has emerged as a pivotal consideration in cancer management. To date, only a limited number of pharmacological agents, including N-acetylcysteine (NAC) (24), silymarin, ursodeoxycholic acid, and tiopronin (25–27), have been investigated for their potential to mitigate DILI, yet their efficacy remains a subject of debate. In this study, we present findings indicating that MgIG can efficaciously and safely ameliorate DILI in individuals with malignant tumors.

MgIG has emerged as a promising therapeutic approach for the management of inflammatory liver diseases (28). Its mechanism of action may involve the alleviation of oxidative stress within hepatic tissue (29). MgIG has gained approval for the treatment of acute DILI with markedly elevated ALT levels, particularly in cases of acute hepatocellular or mixed-type DILI (13). Furthermore, real-world data analyses from China have demonstrated that MgIG, administered either as monotherapy or in conjunction with other liver-protective regimens, outperforms or is comparable to alternative treatments such as supportive therapy and hormone therapy in managing liver injury associated with novel anti-cancer drugs (30). Notably, during treatment with MgIG, inflammatory biomarkers including interleukin-6 (IL-6) and procalcitonin (PCT) undergo significant changes, further attesting to its therapeutic efficacy (31). In line with previous research, our study revealed that at the 30-day mark, the control group exhibited statistically significant increases in ALT, aspartate aminotransferase (AST), and total bilirubin (TBil) levels compared to the MgIG group (*P* < 0.05). Additionally, at the 60-day follow-up, the control group had a significantly higher incidence of gamma-glutamyltransferase (γ-GGT) elevation than the MgIG group (*P* = 0.005), underscoring MgIG's favorable efficacy in DILI patients with hematological malignancies.

This study does encounter a few considerations that merit attention. Firstly, the 2:1 matching ratio between the MgIG and control groups, while operational, might introduce subtle selection biases, and a 1:1 ratio could potentially offer a more balanced comparison. Secondly, while the study provides valuable insights, it would be further strengthened by a direct comparison with other liver protective agents, like tiopronin, to more definitively ascertain MgIG's comparative efficacy. Additionally, the focus on clinical observations has been instrumental, yet an exploration of MgIG's molecular mechanisms of liver protection could deepen our understanding. Lastly, while the study has delivered significant findings, an analysis of disease-free survival (PFS) and overall survival (OS) between the groups would have added another layer of comprehensiveness to the results.

## Conclusion

MgIG pretreatment shows specific hepatoprotective activity in hematological cancers, particularly suppressing abnormal rises in ALT, AST, and TBiL. Our analysis suggests that prophylactic MgIG administration may attenuate the severity of DILI following antineoplastic therapies across various hematological disorders. Although its efficacy in lowering overall DILI incidence remains inconclusive, longitudinal observation reveals clinically meaningful mitigation of hepatotoxicity severity during specific treatment phases and within select patient subgroups.

## Data Availability

The original contributions presented in the study are included in the article/Supplementary material, further inquiries can be directed to the corresponding author.
