# Supplementary material for: Multicenter study on magnesium isoglycyrrhizinate preventing novel antitumor-induced liver injury in hematological malignancies
Source: Front Med (Lausanne). 2026 Jun 26;13:1759611. doi: 10.3389/fmed.2026.1759611 (PMC13349873; doi:10.3389/fmed.2026.1759611)
Supplement: Supplementary file 1 [file Data_Sheet_1.docx]

**Supplementary Materials**

**Supplementary Tables**

**Supplementary Table 1 Novel anticancer drugs used in this study**

| **Classifications** | **Name of novel anticancer drugs** |
| --- | --- |
| Tyrosine kinase inhibitors | Imatinib, Dasatinib, Nilotinib, Olabarrutinib, Flumatinib, Ibrutinib, Zanubrutinib, Ocalivumab, Gilteritinib, Ruxolitinib. |
| Proteasome inhibitors | Bortezomib, Carfilzomib, Ixazomib. |
| Immunomodulatory drugs | Thalidomide, Lenalidomide, Pomalidomide. |
| Monoclonal antibodies | Daratumumab, Blinatumomab, Sintilimab, Camrelizumab, Tiragolumab, Penpulimab, Rituximab, Obinutuzumab |
| Antibody-drug conjugates | Brentuximab, Vedotin |
| Others | Selinexor, Venetoclax, Ivosidenib, Chidamide |

Note: Per the "Guidelines for Clinical Application of Novel Anticancer Drugs (2022 Edition)" refer to small-molecule targeted therapies and large-molecule monoclonal antibodies.

**Supplementary Table 2 Intergroup comparison of the incidence of liver injuries**

| **Liver injury, n (%)** | **MgIG group (N = 324)** | **Control group (N=182)** | **p-value^2^** |
| --- | --- | --- | --- |
|  |  |  |  |
| d21 | 7 (2.8) | 2 (1.5) | 0.503 |
| 95% CI^1^ | 1.1, 5.8 | 0.2, 5.3 |  |
| Missing | 77 | 48 |  |
| d30 | 6 (3.5) | 3 (3.1) | ＞0.999 |
| 95% CI | 1.3, 7.4 | 0.6, 8.8 |  |
| Missing | 152 | 85 |  |
| d60 | 3 (1.6) | 3 (2.9) | 0.669 |
| 95% CI | 0.3, 4.6 | 0.6, 8.4 |  |
| Missing | 138 | 80 |  |
| Post-hepatoprotective drugs | 13 (4.2) | 7 (4.0) | ＞0.999 |
| 95% CI | 2.3, 7.2 | 1.6, 8.1 |  |
| Missing | 18 | 8 |  |

Data are presented as n (%), percentages are based on non-missing value. CI = Confidence Interval; MgIG: magnesium isoglycyrrhizinate

^1^The exact method was used for calculating 95% confidence interval. ^2^Fisher’s exact test was used to compare the incidence of liver injuries between groups.

**Supplementary Table 3 Intergroup comparison of hepatic injurie severity relevant to liver function.**

| **Hepatic injury pertinent to liver function** | **MgIG group (N = 324)** | **Control group (N=182)** | **p-value** |  |
| --- | --- | --- | --- | --- |
|  |  |  |  |  |
| Overall, n (%) | 147 (48.0) | 116 (66.7) | ＜0.001^2^ |  |
| 95% CI^1^ | 42.3, 53.8 | 59.1, 73.6 |  |  |
| Missing | 18 | 8 |  |  |
| Severity, n (%) |  |  | ＜0.001^3^ |  |
| 1 | 100 (32.7) | 81 (46.6) |  |  |
| 95% CI | 27.5, 38.2 | 39.0, 54.3 |  |  |
| 2 | 33 (10.8) | 24 (13.8) |  |  |
| 95% CI | 7.5, 14.8 | 9.0, 19.8 |  |  |
| 1~2 | 133 (43.5) | 105 (60.3) |  |  |
| 95% CI | 37.8, 49.2 | 52.7, 67.7 |  |  |
| 3 | 14 (4.6) | 10 (5.7) |  |  |
| 95% CI | 2.5, 7.6 | 2.8, 10.3 |  |  |
| 2~3 | 47 (15.4) | 35 (20.1) |  |  |
| 95% CI | 11.5, 19.9 | 14.4, 26.8 |  |  |
| 4 | 0 (0.0) | 1 (0.6) |  |  |
| 95% CI | 0.0, 1.2 | 0.0, 3.2 |  |  |
| 3~4 | 14 (4.6) | 11 (6.3) | 0.402^2^ |  |
| 95% CI | 2.5, 7.6 | 3.2, 11.0 |  |  |

Data are presented as n (%), percentages are based on non-missing value.

^1^The exact method was used for calculating 95% confidence interval. ^2^Fisher’s exact test. ^3^Kruskal-Wallis rank sum test.

Abbreviation: CI = Confidence Interval; MgIG: magnesium isoglycyrrhizinate

**Supplementary Table 4. Stratified Analysis by Chemotherapy Administration according to Occurrence of Liver Function–Related Adverse Events.**

| **Characteristic** | **Without chemotherapy** | | | | **With chemotherapy** | | | |
| --- | --- | --- | --- | --- | --- | --- | --- | --- |
|  | **MgIG Group (N = 28)** | **Control Group (N = 65)** | **Overall**  **(N = 93)** | **p-value** | **MgIG Group (N = 296)** | **Control Group (N = 117)** | **Overall**  **(N = 413)** | **p-value** |
| Occurrence of liver function–related AE, n (%) |  |  |  | 0.334^1^ |  |  |  | 0.001^1^ |
| No | 12 (46.2) | 21 (32.8) | 33 (36.7) |  | 147 (52.5) | 37 (33.6) | 184 (47.2) |  |
| Yes | 14 (53.8) | 43 (67.2) | 57 (63.3) |  | 133 (47.5) | 73 (66.4) | 206 (52.8) |  |
| Missing | 2 | 1 | 3 |  | 16 | 7 | 23 |  |
| Worst grade of liver function–related AE, n (%) |  |  |  | 0.088^2^ |  |  |  | 0.008^2^ |
| Grade 0 | 12 (46.2) | 21 (32.8) | 33 (36.7) |  | 147 (52.5) | 37 (33.6) | 184 (47.2) |  |
| Grade 1 | 11 (42.3) | 25 (39.1) | 36 (40.0) |  | 89 (31.8) | 56 (50.9) | 145 (37.2) |  |
| Grade 2 | 3 (11.5) | 13 (20.3) | 16 (17.8) |  | 30 (10.7) | 11 (10.0) | 41 (10.5) |  |
| Grade 3 | 0 (0.0) | 4 (6.3) | 4 (4.4) |  | 14 (5.0) | 6 (5.5) | 20 (5.1) |  |
| Grade 4 | 0 (0.0) | 1 (1.6) | 1 (1.1) |  | — | — | — |  |
| Missing | 2 | 1 | 3 |  | 16 | 7 | 23 |  |
| Worst grade grouping of liver function–related AE, n (%) | |  |  | 0.135^2^ |  |  |  | 0.002^2^ |
| Grade 0 | 12 (46.2) | 21 (32.8) | 33 (36.7) |  | 147 (52.5) | 37 (33.6) | 184 (47.2) |  |
| Grades 1–2 | 14 (53.8) | 38 (59.4) | 52 (57.8) |  | 119 (42.5) | 67 (60.9) | 186 (47.7) |  |
| Grade 3 | 0 (0.0) | 5 (7.8) | 5 (5.6) |  | 14 (5.0) | 6 (5.5) | 20 (5.1) |  |
| Missing | 2 | 1 | 3 |  | 16 | 7 | 23 |  |
| ≥ Grade 2 liver function–related AE, n (%) |  |  |  | 0.107^1^ |  |  |  | >0.999^1^ |
| No | 23 (88.5) | 46 (71.9) | 69 (76.7) |  | 236 (84.3%) | 93 (84.5) | 329 (84.4) |  |
| Yes | 3 (11.5) | 18 (28.1) | 21 (23.3) |  | 44 (15.7) | 17 (15.5) | 61 (15.6) |  |
| Missing | 2 | 1 | 3 |  | 16 | 7 | 23 |  |
| ≥ Grade 3 liver function–related AE, n (%) |  |  |  | 0.316^1^ |  |  |  | 0.804^1^ |
| No | 26 (100.0) | 59 (92.2) | 85 (94.4) |  | 266 (95.0) | 104 (94.5) | 370 (94.9) |  |
| Yes | 0 (0.0) | 5 (7.8) | 5 (5.6) |  | 14 (5.0) | 6 (5.5) | 20 (5.1) |  |
| Missing | 2 | 1 | 3 |  | 16 | 7 | 23 |  |
| Occurrence of liver function–related AE at Day 21, n (%) | |  |  | 0.075^1^ |  |  |  | 0.029^1^ |
| No | 15 (65.2) | 19 (40.4) | 34 (48.6) |  | 141 (62.7) | 42 (48.3) | 183 (58.7) |  |
| Yes | 8 (34.8) | 28 (59.6) | 36 (51.4) |  | 84 (37.3) | 45 (51.7) | 129 (41.3) |  |
| Missing | 5 | 18 | 23 |  | 71 | 30 | 101 |  |
| Worst grade of liver function–related AE at Day 21, n (%) | |  |  | 0.063^2^ |  |  |  | 0.132^2^ |
| Grade 0 | 15 (65.2) | 19 (40.4) | 34 (48.6) |  | 141 (62.7) | 42 (48.3) | 183 (58.7) |  |
| Grade 1 | 6 (26.1) | 21 (44.7) | 27 (38.6) |  | 55 (24.4) | 40 (46.0) | 95 (30.4) |  |
| Grade 2 | 2 (8.7) | 6 (12.8) | 8 (11.4) |  | 20 (8.9) | 5 (5.7) | 25 (8.0) |  |
| Grade 3 | 0 (0.0) | 1 (2.1) | 1 (1.4) |  | 9 (4.0) | 0 (0.0) | 9 (2.9) |  |
| Missing | 5 | 18 | 23 |  | 71 | 30 | 101 |  |
| Worst grade grouping of liver function–related AE at Day 21, n (%) | |  |  | 0.048^2^ |  |  |  | 0.050^2^ |
| Grade 0 | 15 (65.2) | 19 (40.4) | 34 (48.6) |  | 141 (62.7) | 42 (48.3) | 183 (58.7) |  |
| Grades 1–2 | 8 (34.8) | 27 (57.4) | 35 (50.0) |  | 75 (33.3) | 45 (51.7) | 120 (38.5) |  |
| Grade 3 | 0 (0.0) | 1 (2.1) | 1 (1.4) |  | 9 (4.0) | 0 (0.0) | 9 (2.9) |  |
| Missing | 5 | 18 | 23 |  | 71 | 30 | 101 |  |
| ≥ Grade 3 liver function–related AE at Day 21, n (%) |  |  |  | >0.999^1^ |  |  |  | 0.067^1^ |
| No | 23 (100.0) | 46 (97.9) | 69 (98.6) |  | 216 (96.0) | 87 (100.0) | 303 (97.1) |  |
| Yes | 0 (0.0) | 1 (2.1) | 1 (1.4) |  | 9 (4.0) | 0 (0.0) | 9 (2.9) |  |
| Missing | 5 | 18 | 23 |  | 71 | 30 | 101 |  |
| ≥ Grade 2 liver function–related AE at Day 21, n (%) |  |  |  | 0.708^1^ |  |  |  | 0.103^1^ |
| No | 21 (91.3) | 40 (85.1) | 61 (87.1) |  | 196 (87.1) | 82 (94.3) | 278 (89.1) |  |
| Yes | 2 (8.7) | 7 (14.9) | 9 (12.9) |  | 29 (12.9) | 5 (5.7) | 34 (10.9) |  |
| Missing | 5 | 18 | 23 |  | 71 | 30 | 101 |  |
| Occurrence of liver function–related AE at Day 30, n (%) | |  |  | >0.999^1^ |  |  |  | 0.002^1^ |
| No | 7 (43.8) | 10 (40.0) | 17 (41.5) |  | 107 (68.2) | 33 (45.8) | 140 (61.1) |  |
| Yes | 9 (56.3) | 15 (60.0) | 24 (58.5) |  | 50 (31.8) | 39 (54.2) | 89 (38.9) |  |
| Missing | 12 | 40 | 52 |  | 139 | 45 | 184 |  |
| Worst grade of liver function–related AE at Day 30, n (%) | |  |  | 0.337^2^ |  |  |  | 0.004^2^ |
| Grade 0 | 7 (43.8) | 10 (40.0) | 17 (41.5) |  | 107 (68.2) | 33 (45.8) | 140 (61.1) |  |
| Grade 1 | 8 (50.0) | 8 (32.0) | 16 (39.0) |  | 37 (23.6) | 33 (45.8) | 70 (30.6) |  |
| Grade 2 | 1 (6.3) | 3 (12.0) | 4 (9.8) |  | 8 (5.1) | 3 (4.2) | 11 (4.8) |  |
| Grade 3 | 0 (0.0) | 3 (12.0) | 3 (7.3) |  | 5 (3.2) | 3 (4.2) | 8 (3.5) |  |
| Grade 4 | 0 (0.0) | 1 (4.0) | 1 (2.4) |  | — | — | — |  |
| Missing | 12 | 40 | 52 |  | 139 | 45 | 184 |  |
| Worst grade grouping of liver function–related AE at Day 30, n (%) | |  |  | 0.450^2^ |  |  |  | 0.002^2^ |
| Grade 0 | 7 (43.8) | 10 (40.0) | 17 (41.5) |  | 107 (68.2) | 33 (45.8) | 140 (61.1) |  |
| Grades 1–2 | 9 (56.3) | 11 (44.0) | 20 (48.8) |  | 45 (28.7) | 36 (50.0) | 81 (35.4) |  |
| Grade 3 | 0 (0.0) | 4 (16.0) | 4 (9.8) |  | 5 (3.2) | 3 (4.2) | 8 (3.5) |  |
| Missing | 12 | 40 | 52 |  | 139 | 45 | 184 |  |
| ≥ Grade 3 liver function–related AE at Day 30, n (%) |  |  |  | 0.143^1^ |  |  |  | 0.709^1^ |
| No | 16 (100.0) | 21 (84.0) | 37 (90.2) |  | 152 (96.8) | 69 (95.8) | 221 (96.5) |  |
| Yes | 0 (0.0) | 4 (16.0) | 4 (9.8) |  | 5 (3.2) | 3 (4.2) | 8 (3.5) |  |
| Missing | 12 | 40 | 52 |  | 139 | 45 | 184 |  |
| ≥ Grade 2 liver function–related AE at Day 30, n (%) |  |  |  | 0.120^1^ |  |  |  | >0.999^1^ |
| No | 15 (93.8) | 18 (72.0) | 33 (80.5) |  | 144 (91.7) | 66 (91.7) | 210 (91.7) |  |
| Yes | 1 (6.3) | 7 (28.0) | 8 (19.5) |  | 13 (8.3) | 6 (8.3) | 19 (8.3) |  |
| Missing | 12 | 40 | 52 |  | 139 | 45 | 184 |  |
| Occurrence of liver function–related AE at Day 60, n (%) | |  |  | 0.238^1^ |  |  |  | 0.678^1^ |
| No | 13 (65.0) | 11 (45.8) | 24 (54.5) |  | 97 (58.4) | 43 (55.1) | 140 (57.4) |  |
| Yes | 7 (35.0) | 13 (54.2) | 20 (45.5) |  | 69 (41.6) | 35 (44.9) | 104 (42.6) |  |
| Missing | 8 | 41 | 49 |  | 130 | 39 | 169 |  |
| Worst grade of liver function–related AE at Day 60, n (%) | |  |  | 0.121^2^ |  |  |  | 0.400^2^ |
| Grade 0 | 13 (65.0) | 11 (45.8) | 24 (54.5) |  | 97 (58.4) | 43 (55.1) | 140 (57.4) |  |
| Grade 1 | 5 (25.0) | 6 (25.0) | 11 (25.0) |  | 55 (33.1) | 23 (29.5) | 78 (32.0) |  |
| Grade 2 | 2 (10.0) | 5 (20.8) | 7 (15.9) |  | 8 (4.8) | 7 (9.0) | 15 (6.1) |  |
| Grade 3 | 0 (0.0) | 2 (8.3) | 2 (4.5) |  | 6 (3.6) | 5 (6.4) | 11 (4.5) |  |
| Missing | 8 | 41 | 49 |  | 130 | 39 | 169 |  |
| Worst grade grouping of liver function–related AE at Day 60, n (%) | |  |  | 0.154^2^ |  |  |  | 0.529^2^ |
| Grade 0 | 13 (65.0) | 11 (45.8) | 24 (54.5) |  | 97 (58.4) | 43 (55.1) | 140 (57.4) |  |
| Grades 1–2 | 7 (35.0) | 11 (45.8) | 18 (40.9) |  | 63 (38.0) | 30 (38.5) | 93 (38.1) |  |
| Grade 3 | 0 (0.0) | 2 (8.3) | 2 (4.5) |  | 6 (3.6) | 5 (6.4) | 11 (4.5) |  |
| Missing | 8 | 41 | 49 |  | 130 | 39 | 169 |  |
| ≥ Grade 3 liver function–related AE at Day 60, n (%) |  |  |  | 0.493^1^ |  |  |  | 0.336^1^ |
| No | 20 (100.0) | 22 (91.7) | 42 (95.5) |  | 160 (96.4) | 73 (93.6) | 233 (95.5) |  |
| Yes | 0 (0.0) | 2 (8.3) | 2 (4.5) |  | 6 (3.6) | 5 (6.4) | 11 (4.5) |  |
| Missing | 8 | 41 | 49 |  | 130 | 39 | 169 |  |
| ≥ Grade 2 liver function–related AE at Day 60, n (%) |  |  |  | 0.150^1^ |  |  |  | 0.120^1^ |
| No | 18 (90.0) | 17 (70.8) | 35 (79.5) |  | 152 (91.6) | 66 (84.6) | 218 (89.3) |  |
| Yes | 2 (10.0) | 7 (29.2) | 9 (20.5) |  | 14 (8.4) | 12 (15.4) | 26 (10.7) |  |
| Missing | 8 | 41 | 49 |  | 130 | 39 | 169 |  |

Data are presented as n (%), percentages are based on non-missing value.

^1^Fisher’s exact test. ^2^Kruskal-Wallis rank sum test.

Abbreviation: MgIG: magnesium isoglycyrrhizinate

**Supplementary Table 5 Stratified Analysis Hematological Malignancy Type according to Occurrence of Liver Function–Related Adverse Events**

|  | **Lymphoma** | | | | **Myeloma** | | | | **Leukemia** | | | |
| --- | --- | --- | --- | --- | --- | --- | --- | --- | --- | --- | --- | --- |
| **Characteristic** | **MgIG Group (N = 110)** | **Control Group (N = 71)** | **Overall (N = 181)** | **p-value** | **MgIG Group (N = 93)** | **Control Group (N = 46)** | **Overall (N = 139)** | **p-value** | **MgIG Group (N = 121)** | **Control Group (N = 65)** | **Overall (N = 186)** | **p-value** |
| Occurrence of Liver Function-Related AE, n (%) |  |  |  | 0.121^1^ |  |  |  | 0.354^1^ |  |  |  | <0.001^1^ |
| No | 52 (50.5) | 26 (38.2) | 78 (45.6) |  | 43 (48.9) | 17 (38.6) | 60 (45.5) |  | 64 (55.7) | 15 (24.2) | 79 (44.6) |  |
| Yes | 51 (49.5) | 42 (61.8) | 93 (54.4) |  | 45 (51.1) | 27 (61.4) | 72 (54.5) |  | 51 (44.3) | 47 (75.8) | 98 (55.4) |  |
| Missing | 7 | 3 | 10 |  | 5 | 2 | 7 |  | 6 | 3 | 9 |  |
| Worst Grade of Liver Function-Related AE, n (%) |  |  |  | 0.131^2^ |  |  |  | 0.330^2^ |  |  |  | <0.001^2^ |
| Grade 0 | 52 (50.5) | 26 (38.2) | 78 (45.6) |  | 43 (48.9) | 17 (38.6) | 60 (45.5) |  | 64 (55.7) | 15 (24.2) | 79 (44.6) |  |
| Grade 1 | 39 (37.9) | 32 (47.1) | 71 (41.5) |  | 32 (36.4) | 20 (45.5) | 52 (39.4) |  | 29 (25.2) | 29 (46.8) | 58 (32.8) |  |
| Grade 2 | 9 (8.7) | 7 (10.3) | 16 (9.4) |  | 9 (10.2) | 4 (9.1) | 13 (9.8) |  | 15 (13.0) | 13 (21.0) | 28 (15.8) |  |
| Grade 3 | 3 (2.9) | 2 (2.9) | 5 (2.9) |  | 4 (4.5) | 3 (6.8) | 7 (5.3) |  | 7 (6.1) | 5 (8.1) | 12 (6.8) |  |
| Grade 4 | 0 (0.0) | 1 (1.5) | 1 (0.6) |  |  |  |  |  |  |  |  |  |
| Missing | 7 | 3 | 10 |  | 5 | 2 | 7 |  | 6 | 3 | 9 |  |
| Worst Grade Grouping of Liver Function-Related AE, n (%) |  |  |  | 0.112^2^ |  |  |  | 0.250^2^ |  |  |  | <0.001^2^ |
| Grade 0 | 52 (50.5) | 26 (38.2) | 78 (45.6) |  | 43 (48.9) | 17 (38.6) | 60 (45.5) |  | 64 (55.7) | 15 (24.2) | 79 (44.6) |  |
| Grades 1–2 | 48 (46.6) | 39 (57.4) | 87 (50.9) |  | 41 (46.6) | 24 (54.5) | 65 (49.2) |  | 44 (38.3) | 42 (67.7) | 86 (48.6) |  |
| Grade 3 | 3 (2.9) | 3 (4.4) | 6 (3.5) |  | 4 (4.5) | 3 (6.8) | 7 (5.3) |  | 7 (6.1) | 5 (8.1) | 12 (6.8) |  |
| Missing | 7 | 3 | 10 |  | 5 | 2 | 7 |  | 6 | 3 | 9 |  |
| Occurrence of ≥ Grade 2 Liver Function-Related AE, n (%) |  |  |  | 0.643^1^ |  |  |  | >0.999^1^ |  |  |  | 0.138^1^ |
| No | 91 (88.3) | 58 (85.3) | 149 (87.1) |  | 75 (85.2) | 37 (84.1) | 112 (84.8) |  | 93 (80.9) | 44 (71.0) | 137 (77.4) |  |
| Yes | 12 (11.7) | 10 (14.7) | 22 (12.9) |  | 13 (14.8) | 7 (15.9) | 20 (15.2) |  | 22 (19.1) | 18 (29.0) | 40 (22.6) |  |
| Missing | 7 | 3 | 10 |  | 5 | 2 | 7 |  | 6 | 3 | 9 |  |
| Occurrence of ≥ Grade 3 Liver Function-Related AE, n (%) |  |  |  | 0.683^1^ |  |  |  | 0.686^1^ |  |  |  | 0.755^1^ |
| No | 100 (97.1) | 65 (95.6) | 165 (96.5) |  | 84 (95.5) | 41 (93.2) | 125 (94.7) |  | 108 (93.9) | 57 (91.9) | 165 (93.2) |  |
| Yes | 3 (2.9) | 3 (4.4) | 6 (3.5) |  | 4 (4.5) | 3 (6.8) | 7 (5.3) |  | 7 (6.1) | 5 (8.1) | 12 (6.8) |  |
| Missing | 7 | 3 | 10 |  | 5 | 2 | 7 |  | 6 | 3 | 9 |  |
| Occurrence of Liver Function-Related AE at Day 21, n (%) |  |  |  | 0.350^1^ |  |  |  | 0.088^1^ |  |  |  | 0.016^1^ |
| No | 51 (62.2) | 24 (52.2) | 75 (58.6) |  | 49 (63.6) | 14 (43.8) | 63 (57.8) |  | 56 (62.9) | 23 (41.1) | 79 (54.5) |  |
| Yes | 31 (37.8) | 22 (47.8) | 53 (41.4) |  | 28 (36.4) | 18 (56.3) | 46 (42.2) |  | 33 (37.1) | 33 (58.9) | 66 (45.5) |  |
| Missing | 28 | 25 | 53 |  | 16 | 14 | 30 |  | 32 | 9 | 41 |  |
| Worst Grade of Liver Function-Related AE at Day 21, n (%) |  |  |  | 0.408^2^ |  |  |  | 0.114^2^ |  |  |  | 0.077^2^ |
| Grade 0 | 51 (62.2) | 24 (52.2) | 75 (58.6) |  | 49 (63.6) | 14 (43.8) | 63 (57.8) |  | 56 (62.9) | 23 (41.1) | 79 (54.5) |  |
| Grade 1 | 22 (26.8) | 18 (39.1) | 40 (31.3) |  |  |  |  |  |  |  |  |  |

Data are presented as n (%), percentages are based on non-missing value.

^1^Fisher’s exact test. ^2^Kruskal-Wallis rank sum test.

Abbreviation: MgIG: magnesium isoglycyrrhizinate

# Supplementary Table 6. Stratified Analysis by age according to Occurrence of Liver Function–Related Adverse Events.

|  | **<60** | | | | **≥60** | | | |
| --- | --- | --- | --- | --- | --- | --- | --- | --- |
| **Characteristic** | **MgIG Group  (N = 151)** | **Control Group  (N = 81)** | **Overall (N = 232)** | **p-value** | **MgIG Group  (N = 173)** | **Control Group  (N = 101)** | **Overall (N = 274)** | **p-value** |
| Occurrence of Liver Function-Related AE, n (%) |  |  |  | 0.010^1^ |  |  |  | 0.003^1^ |
| No | 69 (47.6) | 23 (29.5) | 92 (41.3) |  | 90 (55.9) | 35 (36.5) | 125 (48.6) |  |
| Yes | 76 (52.4) | 55 (70.5) | 131 (58.7) |  | 71 (44.1) | 61 (63.5) | 132 (51.4) |  |
| Missing | 6 | 3 | 9 |  | 12 | 5 | 17 |  |
| Worst Grade of Liver Function-Related AE, n (%) |  |  |  | 0.029^2^ |  |  |  | 0.004^2^ |
| 0 | 69 (47.6) | 23 (29.5) | 92 (41.3) |  | 90 (55.9) | 35 (36.5) | 125 (48.6) |  |
| 1 | 49 (33.8) | 37 (47.4) | 86 (38.6) |  | 51 (31.7) | 44 (45.8) | 95 (37.0) |  |
| 2 | 18 (12.4) | 14 (17.9) | 32 (14.3) |  | 15 (9.3) | 10 (10.4) | 25 (9.7) |  |
| 3 | 9 (6.2) | 4 (5.1) | 13 (5.8) |  | 5 (3.1) | 6 (6.3) | 11 (4.3) |  |
| 4 |  |  |  |  | 0 (0.0) | 1 (1.0) | 1 (0.4) |  |
| Missing | 6 | 3 | 9 |  | 12 | 5 | 17 |  |
| Worst Grade Grouping of Liver Function-Related AE, n (%) |  |  |  | 0.022^2^ |  |  |  | 0.002^2^ |
| 0 | 69 (47.6) | 23 (29.5) | 92 (41.3) |  | 90 (55.9) | 35 (36.5) | 125 (48.6) |  |
| 1-2 | 67 (46.2) | 51 (65.4) | 118 (52.9) |  | 66 (41.0) | 54 (56.3) | 120 (46.7) |  |
| 3 | 9 (6.2) | 4 (5.1) | 13 (5.8) |  | 5 (3.1) | 7 (7.3) | 12 (4.7) |  |
| Missing | 6 | 3 | 9 |  | 12 | 5 | 17 |  |
| Occurrence of ≥ Grade 2 Liver Function-Related AE, n (%) |  |  |  | 0.485^1^ |  |  |  | 0.272^1^ |
| No | 118 (81.4) | 60 (76.9) | 178 (79.8) |  | 141 (87.6) | 79 (82.3) | 220 (85.6) |  |
| Yes | 27 (18.6) | 18 (23.1) | 45 (20.2) |  | 20 (12.4) | 17 (17.7) | 37 (14.4) |  |
| Missing | 6 | 3 | 9 |  | 12 | 5 | 17 |  |
| Occurrence of ≥ Grade 3 Liver Function-Related AE, n (%) |  |  |  | >0.999^1^ |  |  |  | 0.137^1^ |
| No | 136 (93.8) | 74 (94.9) | 210 (94.2) |  | 156 (96.9) | 89 (92.7) | 245 (95.3) |  |
| Yes | 9 (6.2) | 4 (5.1) | 13 (5.8) |  | 5 (3.1) | 7 (7.3) | 12 (4.7) |  |
| Missing | 6 | 3 | 9 |  | 12 | 5 | 17 |  |
| Occurrence of Liver Function-Related AE at Day 21, n (%) |  |  |  | 0.018^1^ |  |  |  | 0.037^1^ |
| No | 73 (61.3) | 26 (41.9) | 99 (54.7) |  | 83 (64.3) | 35 (48.6) | 118 (58.7) |  |
| Yes | 46 (38.7) | 36 (58.1) | 82 (45.3) |  | 46 (35.7) | 37 (51.4) | 83 (41.3) |  |
| Missing | 32 | 19 | 51 |  | 44 | 29 | 73 |  |
| Worst Grade Grouping of Liver Function-Related AE at Day 21, n (%) |  |  |  | 0.053^2^ |  |  |  | 0.107^2^ |
| 0 | 73 (61.3) | 26 (41.9) | 99 (54.7) |  | 83 (64.3) | 35 (48.6) | 118 (58.7) |  |
| 1 | 30 (25.2) | 29 (46.8) | 59 (32.6) |  | 31 (24.0) | 32 (44.4) | 63 (31.3) |  |
| 2 | 11 (9.2) | 6 (9.7) | 17 (9.4) |  | 11 (8.5) | 5 (6.9) | 16 (8.0) |  |
| 3 | 5 (4.2) | 1 (1.6) | 6 (3.3) |  | 4 (3.1) | 0 (0.0) | 4 (2.0) |  |
| Missing | 32 | 19 | 51 |  | 44 | 29 | 73 |  |
| Worst Grade Grouping of Liver Function-Related AE at Day 21, n (%) |  |  |  | 0.026^2^ |  |  |  | 0.053^2^ |
| 0 | 73 (61.3) | 26 (41.9) | 99 (54.7) |  | 83 (64.3) | 35 (48.6) | 118 (58.7) |  |
| 1-2 | 41 (34.5) | 35 (56.5) | 76 (42.0) |  | 42 (32.6) | 37 (51.4) | 79 (39.3) |  |
| 3 | 5 (4.2) | 1 (1.6) | 6 (3.3) |  | 4 (3.1) | 0 (0.0) | 4 (2.0) |  |
| Missing | 32 | 19 | 51 |  | 44 | 29 | 73 |  |
| Occurrence of ≥ Grade 3 Liver Function-Related AE at Day 21, n (%) |  |  |  | 0.666^1^ |  |  |  | 0.299^1^ |
| No | 114 (95.8) | 61 (98.4) | 175 (96.7) |  | 125 (96.9) | 72 (100.0) | 197 (98.0) |  |
| Yes | 5 (4.2) | 1 (1.6) | 6 (3.3) |  | 4 (3.1) | 0 (0.0) | 4 (2.0) |  |
| Missing | 32 | 19 | 51 |  | 44 | 29 | 73 |  |
| Occurrence of ≥ Grade 2 Liver Function-Related AE at Day 21, n (%) |  |  |  | 0.816^1^ |  |  |  | 0.335^1^ |
| No | 103 (86.6) | 55 (88.7) | 158 (87.3) |  | 114 (88.4) | 67 (93.1) | 181 (90.0) |  |
| Yes | 16 (13.4) | 7 (11.3) | 23 (12.7) |  | 15 (11.6) | 5 (6.9) | 20 (10.0) |  |
| Missing | 32 | 19 | 51 |  | 44 | 29 | 73 |  |
| Occurrence of Liver Function-Related AE at Day 30, n (%) |  |  |  | 0.041^1^ |  |  |  | 0.018^1^ |
| No | 45 (60.0) | 19 (39.6) | 64 (52.0) |  | 69 (70.4) | 24 (49.0) | 93 (63.3) |  |
| Yes | 30 (40.0) | 29 (60.4) | 59 (48.0) |  | 29 (29.6) | 25 (51.0) | 54 (36.7) |  |
| Missing | 76 | 33 | 109 |  | 75 | 52 | 127 |  |
| Worst Grade of Liver Function-Related AE at Day 30, n (%) |  |  |  | 0.027^2^ |  |  |  | 0.014^2^ |
| 0 | 45 (60.0) | 19 (39.6) | 64 (52.0) |  | 69 (70.4) | 24 (49.0) | 93 (63.3) |  |
| 1 | 23 (30.7) | 21 (43.8) | 44 (35.8) |  | 22 (22.4) | 20 (40.8) | 42 (28.6) |  |
| 2 | 4 (5.3) | 5 (10.4) | 9 (7.3) |  | 5 (5.1) | 1 (2.0) | 6 (4.1) |  |
| 3 | 3 (4.0) | 3 (6.3) | 6 (4.9) |  | 2 (2.0) | 3 (6.1) | 5 (3.4) |  |
| 4 |  |  |  |  | 0 (0.0) | 1 (2.0) | 1 (0.7) |  |
| Missing | 76 | 33 | 109 |  | 75 | 52 | 127 |  |
| Worst Grade Grouping of Liver Function-Related AE at Day 30, n (%) |  |  |  | 0.030^2^ |  |  |  | 0.008^2^ |
| 0 | 45 (60.0) | 19 (39.6) | 64 (52.0) |  | 69 (70.4) | 24 (49.0) | 93 (63.3) |  |
| 1-2 | 27 (36.0) | 26 (54.2) | 53 (43.1) |  | 27 (27.6) | 21 (42.9) | 48 (32.7) |  |
| 3 | 3 (4.0) | 3 (6.3) | 6 (4.9) |  | 2 (2.0) | 4 (8.2) | 6 (4.1) |  |
| Missing | 76 | 33 | 109 |  | 75 | 52 | 127 |  |
| Occurrence of ≥ Grade 3 Liver Function-Related AE at Day 30, n (%) |  |  |  | 0.677^1^ |  |  |  | 0.096^1^ |
| No | 72 (96.0) | 45 (93.8) | 117 (95.1) |  | 96 (98.0) | 45 (91.8) | 141 (95.9) |  |
| Yes | 3 (4.0) | 3 (6.3) | 6 (4.9) |  | 2 (2.0) | 4 (8.2) | 6 (4.1) |  |
| Missing | 76 | 33 | 109 |  | 75 | 52 | 127 |  |
| Occurrence of ≥ Grade 2 Liver Function-Related AE at Day 30, n (%) |  |  |  | 0.265^1^ |  |  |  | 0.535^1^ |
| No | 68 (90.7) | 40 (83.3) | 108 (87.8) |  | 91 (92.9) | 44 (89.8) | 135 (91.8) |  |
| Yes | 7 (9.3) | 8 (16.7) | 15 (12.2) |  | 7 (7.1) | 5 (10.2) | 12 (8.2) |  |
| Missing | 76 | 33 | 109 |  | 75 | 52 | 127 |  |
| Occurrence of Liver Function-Related AE at Day 60, n (%) |  |  |  | 0.582^1^ |  |  |  | 0.502^1^ |
| No | 51 (58.6) | 24 (52.2) | 75 (56.4) |  | 59 (59.6) | 30 (53.6) | 89 (57.4) |  |
| Yes | 36 (41.4) | 22 (47.8) | 58 (43.6) |  | 40 (40.4) | 26 (46.4) | 66 (42.6) |  |
| Missing | 64 | 35 | 99 |  | 74 | 45 | 119 |  |
| Worst Grade of Liver Function-Related AE at Day 60, n (%) |  |  |  | 0.372^2^ |  |  |  | 0.201^2^ |
| 0 | 51 (58.6) | 24 (52.2) | 75 (56.4) |  | 59 (59.6) | 30 (53.6) | 89 (57.4) |  |
| 1 | 25 (28.7) | 13 (28.3) | 38 (28.6) |  | 35 (35.4) | 16 (28.6) | 51 (32.9) |  |
| 2 | 7 (8.0) | 6 (13.0) | 13 (9.8) |  | 3 (3.0) | 6 (10.7) | 9 (5.8) |  |
| 3 | 4 (4.6) | 3 (6.5) | 7 (5.3) |  | 2 (2.0) | 4 (7.1) | 6 (3.9) |  |
| Missing | 64 | 35 | 99 |  | 74 | 45 | 119 |  |
| Worst Grade Grouping of Liver Function-Related AE at Day 60, n (%) |  |  |  | 0.452^2^ |  |  |  | 0.343^2^ |
| 0 | 51 (58.6) | 24 (52.2) | 75 (56.4) |  | 59 (59.6) | 30 (53.6) | 89 (57.4) |  |
| 1-2 | 32 (36.8) | 19 (41.3) | 51 (38.3) |  | 38 (38.4) | 22 (39.3) | 60 (38.7) |  |
| 3 | 4 (4.6) | 3 (6.5) | 7 (5.3) |  | 2 (2.0) | 4 (7.1) | 6 (3.9) |  |
| Missing | 64 | 35 | 99 |  | 74 | 45 | 119 |  |
| Occurrence of ≥ Grade 3 Liver Function-Related AE at Day 60, n (%) |  |  |  | 0.693^1^ |  |  |  | 0.190^1^ |
| No | 83 (95.4) | 43 (93.5) | 126 (94.7) |  | 97 (98.0) | 52 (92.9) | 149 (96.1) |  |
| Yes | 4 (4.6) | 3 (6.5) | 7 (5.3) |  | 2 (2.0) | 4 (7.1) | 6 (3.9) |  |
| Missing | 64 | 35 | 99 |  | 74 | 45 | 119 |  |
| Occurrence of ≥ Grade 2 Liver Function-Related AE at Day 30, n (%) |  |  |  | 0.315^1^ |  |  |  | 0.020^1^ |
| No | 76 (87.4) | 37 (80.4) | 113 (85.0) |  | 94 (94.9) | 46 (82.1) | 140 (90.3) | 0.003^1^ |
| Yes | 11 (12.6) | 9 (19.6) | 20 (15.0) |  | 5 (5.1) | 10 (17.9) | 15 (9.7) |  |
| Missing | 64 | 35 | 99 |  | 74 | 45 | 119 |  |

Data are presented as n (%), percentages are based on non-missing value.

^1^Fisher’s exact test. ^2^Kruskal-Wallis rank sum test.

Abbreviation: MgIG: magnesium isoglycyrrhizinate

# Supplementary Table 7. Comparison of liver function-related adverse events among groups stratified by age

|  | **MgIG Group** | | | | **Control Group** | | | |
| --- | --- | --- | --- | --- | --- | --- | --- | --- |
| **Characteristic** | **<60**  N = 151 | **≥60**  N = 173 | **Overall (N = 324)** | **p-value** | **<60**  N = 81 | **≥60**  N = 101 | **Overall (N = 182)** | **p-value** |
| Occurrence of liver function-related adverse events, n (%) |  |  |  | 0.169^1^ |  |  |  | 0.419^1^ |
| No | 69 (47.6) | 90 (55.9) | 159 (52.0) |  | 23 (29.5) | 35 (36.5) | 58 (33.3) |  |
| Yes | 76 (52.4) | 71 (44.1) | 147 (48.0) |  | 55 (70.5) | 61 (63.5) | 116 (66.7) |  |
| Missing | 6 | 12 | 18 |  | 3 | 5 | 8 |  |
| Worst grade of liver function-related adverse events, n (%) |  |  |  | 0.089^2^ |  |  |  | 0.309^2^ |
| 0 | 69 (47.6) | 90 (55.9) | 159 (52.0) |  | 23 (29.5) | 35 (36.5) | 58 (33.3) |  |
| 1 | 49 (33.8) | 51 (31.7) | 100 (32.7) |  | 37 (47.4) | 44 (45.8) | 81 (46.6) |  |
| 2 | 18 (12.4) | 15 (9.3) | 33 (10.8) |  | 14 (17.9) | 10 (10.4) | 24 (13.8) |  |
| 3 | 9 (6.2) | 5 (3.1) | 14 (4.6) |  | 4 (5.1) | 6 (6.3) | 10 (5.7) |  |
| 4 |  |  |  |  | 0 (0.0) | 1 (1.0) | 1 (0.6) |  |
| Missing | 6 | 12 | 18 |  | 3 | 5 | 8 |  |
| Stratification of the worst grade of liver function-related adverse events, n (%) |  |  |  | 0.106^2^ |  |  |  | 0.504^2^ |
| 0 | 69 (47.6) | 90 (55.9) | 159 (52.0) |  | 23 (29.5) | 35 (36.5) | 58 (33.3) |  |
| 1-2 | 67 (46.2) | 66 (41.0) | 133 (43.5) |  | 51 (65.4) | 54 (56.3) | 105 (60.3) |  |
| 3 | 9 (6.2) | 5 (3.1) | 14 (4.6) |  | 4 (5.1) | 7 (7.3) | 11 (6.3) |  |
| Missing | 6 | 12 | 18 |  | 3 | 5 | 8 |  |
| Occurrence of grade ≥2 liver function-related adverse events, n (%) |  |  |  | 0.154^1^ |  |  |  | 0.448^1^ |
| No | 118 (81.4) | 141 (87.6) | 259 (84.6) |  | 60 (76.9) | 79 (82.3) | 139 (79.9) |  |
| Yes | 27 (18.6) | 20 (12.4) | 47 (15.4) |  | 18 (23.1) | 17 (17.7) | 35 (20.1) |  |
| Missing | 6 | 12 | 18 |  | 3 | 5 | 8 |  |
| Occurrence of grade ≥3 liver function-related adverse events, n (%) |  |  |  | 0.274^1^ |  |  |  | 0.756^1^ |
| No | 136 (93.8) | 156 (96.9) | 292 (95.4) |  | 74 (94.9) | 89 (92.7) | 163 (93.7) |  |
| Yes | 9 (6.2) | 5 (3.1) | 14 (4.6) |  | 4 (5.1) | 7 (7.3) | 11 (6.3) |  |
| Missing | 6 | 12 | 18 |  | 3 | 5 | 8 |  |
| Occurrence of liver function-related adverse events at Day 21, n (%) |  |  |  | 0.693^1^ |  |  |  | 0.489^1^ |
| No | 73 (61.3) | 83 (64.3) | 156 (62.9) |  | 26 (41.9) | 35 (48.6) | 61 (45.5) |  |
| Yes | 46 (38.7) | 46 (35.7) | 92 (37.1) |  | 36 (58.1) | 37 (51.4) | 73 (54.5) |  |
| Missing | 32 | 44 | 76 |  | 19 | 29 | 48 |  |
| Worst grade of liver function-related adverse events at Day 21, n (%) |  |  |  | 0.593^2^ |  |  |  | 0.344^2^ |
| 0 | 73 (61.3) | 83 (64.3) | 156 (62.9) |  | 26 (41.9) | 35 (48.6) | 61 (45.5) |  |
| 1 | 30 (25.2) | 31 (24.0) | 61 (24.6) |  | 29 (46.8) | 32 (44.4) | 61 (45.5) |  |
| 2 | 11 (9.2) | 11 (8.5) | 22 (8.9) |  | 6 (9.7) | 5 (6.9) | 11 (8.2) |  |
| 3 | 5 (4.2) | 4 (3.1) | 9 (3.6) |  | 1 (1.6) | 0 (0.0) | 1 (0.7) |  |
| Missing | 32 | 44 | 76 |  | 19 | 29 | 48 |  |
| Stratification of the worst grade of liver function-related adverse events at Day 21, n (%) |  |  |  | 0.596^2^ |  |  |  | 0.388^2^ |
| 0 | 73 (61.3) | 83 (64.3) | 156 (62.9) |  | 26 (41.9) | 35 (48.6) | 61 (45.5) |  |
| 1-2 | 41 (34.5) | 42 (32.6) | 83 (33.5) |  | 35 (56.5) | 37 (51.4) | 72 (53.7) |  |
| 3 | 5 (4.2) | 4 (3.1) | 9 (3.6) |  | 1 (1.6) | 0 (0.0) | 1 (0.7) |  |
| Missing | 32 | 44 | 76 |  | 19 | 29 | 48 |  |
| Occurrence of grade ≥3 liver function-related adverse events at Day 21, n (%) |  |  |  | 0.741^1^ |  |  |  | 0.463^1^ |
| No | 114 (95.8) | 125 (96.9) | 239 (96.4) |  | 61 (98.4) | 72 (100.0) | 133 (99.3) |  |
| Yes | 5 (4.2) | 4 (3.1) | 9 (3.6) |  | 1 (1.6) | 0 (0.0) | 1 (0.7) |  |
| Missing | 32 | 44 | 76 |  | 19 | 29 | 48 |  |
| Occurrence of grade ≥2 liver function-related adverse events at Day 21, n (%) |  |  |  | 0.704^1^ |  |  |  | 0.546^1^ |
| No | 103 (86.6) | 114 (88.4) | 217 (87.5) |  | 55 (88.7) | 67 (93.1) | 122 (91.0) |  |
| Yes | 16 (13.4) | 15 (11.6) | 31 (12.5) |  | 7 (11.3) | 5 (6.9) | 12 (9.0) |  |
| Missing | 32 | 44 | 76 |  | 19 | 29 | 48 |  |
| Occurrence of liver function-related adverse events at Day 30, n (%) |  |  |  | 0.195^1^ |  |  |  | 0.416^1^ |
| No | 45 (60.0) | 69 (70.4) | 114 (65.9) |  | 19 (39.6) | 24 (49.0) | 43 (44.3) |  |
| Yes | 30 (40.0) | 29 (29.6) | 59 (34.1) |  | 29 (60.4) | 25 (51.0) | 54 (55.7) |  |
| Missing | 76 | 75 | 151 |  | 33 | 52 | 85 |  |
| Worst grade of liver function-related adverse events at Day 30, n (%) |  |  |  | 0.160^2^ |  |  |  | 0.313^2^ |
| 0 | 45 (60.0) | 69 (70.4) | 114 (65.9) |  | 19 (39.6) | 24 (49.0) | 43 (44.3) |  |
| 1 | 23 (30.7) | 22 (22.4) | 45 (26.0) |  | 21 (43.8) | 20 (40.8) | 41 (42.3) |  |
| 2 | 4 (5.3) | 5 (5.1) | 9 (5.2) |  | 5 (10.4) | 1 (2.0) | 6 (6.2) |  |
| 3 | 3 (4.0) | 2 (2.0) | 5 (2.9) |  | 3 (6.3) | 3 (6.1) | 6 (6.2) |  |
| 4 |  |  |  |  | 0 (0.0) | 1 (2.0) | 1 (1.0) |  |
| Missing | 76 | 75 | 151 |  | 33 | 52 | 85 |  |
| Stratification of the worst grade of liver function-related adverse events at Day 30, n (%) |  |  |  | 0.142^2^ |  |  |  | 0.467^2^ |
| 0 | 45 (60.0) | 69 (70.4) | 114 (65.9) |  | 19 (39.6) | 24 (49.0) | 43 (44.3) |  |
| 1-2 | 27 (36.0) | 27 (27.6) | 54 (31.2) |  | 26 (54.2) | 21 (42.9) | 47 (48.5) |  |
| 3 | 3 (4.0) | 2 (2.0) | 5 (2.9) |  | 3 (6.3) | 4 (8.2) | 7 (7.2) |  |
| Missing | 76 | 75 | 151 |  | 33 | 52 | 85 |  |
| Occurrence of grade ≥3 liver function-related adverse events at Day 30, n (%) |  |  |  | 0.654^1^ |  |  |  | >0.999^1^ |
| No | 72 (96.0) | 96 (98.0) | 168 (97.1) |  | 45 (93.8) | 45 (91.8) | 90 (92.8) |  |
| Yes | 3 (4.0) | 2 (2.0) | 5 (2.9) |  | 3 (6.3) | 4 (8.2) | 7 (7.2) |  |
| Missing | 76 | 75 | 151 |  | 33 | 52 | 85 |  |
| Occurrence of grade ≥2 liver function-related adverse events at Day 30, n (%) |  |  |  | 0.780^1^ |  |  |  | 0.387^1^ |
| No | 68 (90.7) | 91 (92.9) | 159 (91.9) |  | 40 (83.3) | 44 (89.8) | 84 (86.6) |  |
| Yes | 7 (9.3) | 7 (7.1) | 14 (8.1) |  | 8 (16.7) | 5 (10.2) | 13 (13.4) |  |
| Missing | 76 | 75 | 151 |  | 33 | 52 | 85 |  |
| Occurrence of liver function-related adverse events at Day 60, n (%) |  |  |  | >0.999^1^ |  |  |  | >0.999^1^ |
| No | 51 (58.6) | 59 (59.6) | 110 (59.1) |  | 24 (52.2) | 30 (53.6) | 54 (52.9) |  |
| Yes | 36 (41.4) | 40 (40.4) | 76 (40.9) |  | 22 (47.8) | 26 (46.4) | 48 (47.1) |  |
| Missing | 64 | 74 | 138 |  | 35 | 45 | 80 |  |
| Worst grade of liver function-related adverse events at Day 60, n (%) |  |  |  | 0.592^2^ |  |  |  | 0.871^2^ |
| 0 | 51 (58.6) | 59 (59.6) | 110 (59.1) |  | 24 (52.2) | 30 (53.6) | 54 (52.9) |  |
| 1 | 25 (28.7) | 35 (35.4) | 60 (32.3) |  | 13 (28.3) | 16 (28.6) | 29 (28.4) |  |
| 2 | 7 (8.0) | 3 (3.0) | 10 (5.4) |  | 6 (13.0) | 6 (10.7) | 12 (11.8) |  |
| 3 | 4 (4.6) | 2 (2.0) | 6 (3.2) |  | 3 (6.5) | 4 (7.1) | 7 (6.9) |  |
| Missing | 64 | 74 | 138 |  | 35 | 45 | 80 |  |
| Stratification of the worst grade of liver function-related adverse events at Day 60, n (%) |  |  |  | 0.785^2^ |  |  |  | 0.921^2^ |
| 0 | 51 (58.6) | 59 (59.6) | 110 (59.1) |  | 24 (52.2) | 30 (53.6) | 54 (52.9) |  |
| 1-2 | 32 (36.8) | 38 (38.4) | 70 (37.6) |  | 19 (41.3) | 22 (39.3) | 41 (40.2) |  |
| 3 | 4 (4.6) | 2 (2.0) | 6 (3.2) |  | 3 (6.5) | 4 (7.1) | 7 (6.9) |  |
| Missing | 64 | 74 | 138 |  | 35 | 45 | 80 |  |
| Occurrence of grade ≥3 liver function-related adverse events at Day 60, n (%) |  |  |  | 0.421^1^ |  |  |  | >0.999^1^ |
| No | 83 (95.4) | 97 (98.0) | 180 (96.8) |  | 43 (93.5) | 52 (92.9) | 95 (93.1) |  |
| Yes | 4 (4.6) | 2 (2.0) | 6 (3.2) |  | 3 (6.5) | 4 (7.1) | 7 (6.9) |  |
| Missing | 64 | 74 | 138 |  | 35 | 45 | 80 |  |
| Occurrence of grade ≥2 liver function-related adverse events at Day 60, n (%) |  |  |  | 0.073^1^ |  |  |  | >0.999^1^ |
| No | 76 (87.4) | 94 (94.9) | 170 (91.4) |  | 37 (80.4) | 46 (82.1) | 83 (81.4) |  |
| Yes | 11 (12.6) | 5 (5.1) | 16 (8.6) |  | 9 (19.6) | 10 (17.9) | 19 (18.6) |  |
| Missing | 64 | 74 | 138 |  | 35 | 45 | 80 |  |
| Data are presented as n (%), percentages are based on non-missing value.  ^1^Fisher's exact test. ^2^Kruskal-Wallis rank sum test | | | | | | | | |

# Supplementary Table 8. Comparison of liver function-related adverse events among groups stratified by chemotherapy

|  | **MgIG Group** | | | | **Control Group** | | | |
| --- | --- | --- | --- | --- | --- | --- | --- | --- |
| **Characteristic** | **No  N = 28** | **Yes**  **N = 296** | **Overall (N = 324)** | **p-value** | **No  N = 65** | **Yes  N = 117** | **Overall (N = 182)** | **p-value** |
| Occurrence of liver function-related adverse events, n (%) |  |  |  | 0.546^1^ |  |  |  | >0.999^1^ |
| No | 12 (46.2) | 147 (52.5) | 159 (52.0) |  | 21 (32.8) | 37 (33.6) | 58 (33.3) |  |
| Yes | 14 (53.8) | 133 (47.5) | 147 (48.0) |  | 43 (67.2) | 73 (66.4) | 116 (66.7) |  |
| Missing | 2 | 16 | 18 |  | 1 | 7 | 8 |  |
| Worst grade of liver function-related adverse events, n (%) |  |  |  | 0.795^2^ |  |  |  | 0.294^2^ |
| 0 | 12 (46.2) | 147 (52.5) | 159 (52.0) |  | 21 (32.8) | 37 (33.6) | 58 (33.3) |  |
| 1 | 11 (42.3) | 89 (31.8) | 100 (32.7) |  | 25 (39.1) | 56 (50.9) | 81 (46.6) |  |
| 2 | 3 (11.5) | 30 (10.7) | 33 (10.8) |  | 13 (20.3) | 11 (10.0) | 24 (13.8) |  |
| 3 | 0 (0.0) | 14 (5.0) | 14 (4.6) |  | 4 (6.3) | 6 (5.5) | 10 (5.7) |  |
| 4 |  |  |  |  | 1 (1.6) | 0 (0.0) | 1 (0.6) |  |
| Missing | 2 | 16 | 18 |  | 1 | 7 | 8 |  |
| Stratification of the worst grade of liver function-related adverse events, n (%) |  |  |  | 0.727^2^ |  |  |  | 0.765^2^ |
| 0 | 12 (46.2) | 147 (52.5) | 159 (52.0) |  | 21 (32.8) | 37 (33.6) | 58 (33.3) |  |
| 1-2 | 14 (53.8) | 119 (42.5) | 133 (43.5) |  | 38 (59.4) | 67 (60.9) | 105 (60.3) |  |
| 3 | 0 (0.0) | 14 (5.0) | 14 (4.6) |  | 5 (7.8) | 6 (5.5) | 11 (6.3) |  |
| Missing | 2 | 16 | 18 |  | 1 | 7 | 8 |  |
| Occurrence of grade ≥2 liver function-related adverse events, n (%) |  |  |  | 0.778^1^ |  |  |  | 0.051^1^ |
| No | 23 (88.5) | 236 (84.3) | 259 (84.6) |  | 46 (71.9) | 93 (84.5) | 139 (79.9) |  |
| Yes | 3 (11.5) | 44 (15.7) | 47 (15.4) |  | 18 (28.1) | 17 (15.5) | 35 (20.1) |  |
| Missing | 2 | 16 | 18 |  | 1 | 7 | 8 |  |
| Occurrence of grade ≥3 liver function-related adverse events, n (%) |  |  |  | 0.618^1^ |  |  |  | 0.536^1^ |
| No | 26 (100.0) | 266 (95.0) | 292 (95.4) |  | 59 (92.2) | 104 (94.5) | 163 (93.7) |  |
| Yes | 0 (0.0) | 14 (5.0) | 14 (4.6) |  | 5 (7.8) | 6 (5.5) | 11 (6.3) |  |
| Missing | 2 | 16 | 18 |  | 1 | 7 | 8 |  |
| Occurrence of liver function-related adverse events at Day 21, n (%) |  |  |  | >0.999^1^ |  |  |  | 0.468^1^ |
| No | 15 (65.2) | 141 (62.7) | 156 (62.9) |  | 19 (40.4) | 42 (48.3) | 61 (45.5) |  |
| Yes | 8 (34.8) | 84 (37.3) | 92 (37.1) |  | 28 (59.6) | 45 (51.7) | 73 (54.5) |  |
| Missing | 5 | 71 | 76 |  | 18 | 30 | 48 |  |
| Worst grade of liver function-related adverse events at Day 21, n (%) |  |  |  | 0.703^2^ |  |  |  | 0.195^2^ |
| 0 | 15 (65.2) | 141 (62.7) | 156 (62.9) |  | 19 (40.4) | 42 (48.3) | 61 (45.5) |  |
| 1 | 6 (26.1) | 55 (24.4) | 61 (24.6) |  | 21 (44.7) | 40 (46.0) | 61 (45.5) |  |
| 2 | 2 (8.7) | 20 (8.9) | 22 (8.9) |  | 6 (12.8) | 5 (5.7) | 11 (8.2) |  |
| 3 | 0 (0.0) | 9 (4.0) | 9 (3.6) |  | 1 (2.1) | 0 (0.0) | 1 (0.7) |  |
| Missing | 5 | 71 | 76 |  | 18 | 30 | 48 |  |
| Stratification of the worst grade of liver function-related adverse events at Day 21, n (%) |  |  |  | 0.713^2^ |  |  |  | 0.325^2^ |
| 0 | 15 (65.2) | 141 (62.7) | 156 (62.9) |  | 19 (40.4) | 42 (48.3) | 61 (45.5) |  |
| 1-2 | 8 (34.8) | 75 (33.3) | 83 (33.5) |  | 27 (57.4) | 45 (51.7) | 72 (53.7) |  |
| 3 | 0 (0.0) | 9 (4.0) | 9 (3.6) |  | 1 (2.1) | 0 (0.0) | 1 (0.7) |  |
| Missing | 5 | 71 | 76 |  | 18 | 30 | 48 |  |
| Occurrence of grade ≥3 liver function-related adverse events at Day 21, n (%) |  |  |  | >0.999^1^ |  |  |  | 0.351^1^ |
| No | 23 (100.0) | 216 (96.0) | 239 (96.4) |  | 46 (97.9) | 87 (100.0) | 133 (99.3) |  |
| Yes | 0 (0.0) | 9 (4.0) | 9 (3.6) |  | 1 (2.1) | 0 (0.0) | 1 (0.7) |  |
| Missing | 5 | 71 | 76 |  | 18 | 30 | 48 |  |
| Occurrence of grade ≥2 liver function-related adverse events at Day 21, n (%) |  |  |  | 0.748^1^ |  |  |  | 0.111^1^ |
| No | 21 (91.3) | 196 (87.1) | 217 (87.5) |  | 40 (85.1) | 82 (94.3) | 122 (91.0) |  |
| Yes | 2 (8.7) | 29 (12.9) | 31 (12.5) |  | 7 (14.9) | 5 (5.7) | 12 (9.0) |  |
| Missing | 5 | 71 | 76 |  | 18 | 30 | 48 |  |
| Occurrence of liver function-related adverse events at Day 30, n (%) |  |  |  | 0.058^1^ |  |  |  | 0.648^1^ |
| No | 7 (43.8) | 107 (68.2) | 114 (65.9) |  | 10 (40.0) | 33 (45.8) | 43 (44.3) |  |
| Yes | 9 (56.3) | 50 (31.8) | 59 (34.1) |  | 15 (60.0) | 39 (54.2) | 54 (55.7) |  |
| Missing | 12 | 139 | 151 |  | 40 | 45 | 85 |  |
| Worst grade of liver function-related adverse events at Day 30, (%) |  |  |  | 0.089^2^ |  |  |  | 0.185^2^ |
| 0 | 7 (43.8) | 107 (68.2) | 114 (65.9) |  | 10 (40.0) | 33 (45.8) | 43 (44.3) |  |
| 1 | 8 (50.0) | 37 (23.6) | 45 (26.0) |  | 8 (32.0) | 33 (45.8) | 41 (42.3) |  |
| 2 | 1 (6.3) | 8 (5.1) | 9 (5.2) |  | 3 (12.0) | 3 (4.2) | 6 (6.2) |  |
| 3 | 0 (0.0) | 5 (3.2) | 5 (2.9) |  | 3 (12.0) | 3 (4.2) | 6 (6.2) |  |
| 4 |  |  |  |  | 1 (4.0) | 0 (0.0) | 1 (1.0) |  |
| Missing | 12 | 139 | 151 |  | 40 | 45 | 85 |  |
| Stratification of the worst grade of liver function-related adverse events at Day 30, n (%) |  |  |  | 0.072^2^ |  |  |  | 0.319^2^ |
| 0 | 7 (43.8) | 107 (68.2) | 114 (65.9) |  | 10 (40.0) | 33 (45.8) | 43 (44.3) |  |
| 1-2 | 9 (56.3) | 45 (28.7) | 54 (31.2) |  | 11 (44.0) | 36 (50.0) | 47 (48.5) |  |
| 3 | 0 (0.0) | 5 (3.2) | 5 (2.9) |  | 4 (16.0) | 3 (4.2) | 7 (7.2) |  |
| Missing | 12 | 139 | 151 |  | 40 | 45 | 85 |  |
| Occurrence of grade ≥3 liver function-related adverse events at Day 30, n (%) |  |  |  | >0.999^1^ |  |  |  | 0.070^1^ |
| No | 16 (100.0) | 152 (96.8) | 168 (97.1) |  | 21 (84.0) | 69 (95.8) | 90 (92.8) |  |
| Yes | 0 (0.0) | 5 (3.2) | 5 (2.9) |  | 4 (16.0) | 3 (4.2) | 7 (7.2) |  |
| Missing | 12 | 139 | 151 |  | 40 | 45 | 85 |  |
| Occurrence of grade ≥2 liver function-related adverse events at Day 30, n (%) |  |  |  | >0.999^1^ |  |  |  | 0.035^1^ |
| No | 15 (93.8) | 144 (91.7) | 159 (91.9) |  | 18 (72.0) | 66 (91.7) | 84 (86.6) |  |
| Yes | 1 (6.3) | 13 (8.3) | 14 (8.1) |  | 7 (28.0) | 6 (8.3) | 13 (13.4) |  |
| Missing | 12 | 139 | 151 |  | 40 | 45 | 85 |  |
| Occurrence of liver function-related adverse events at Day 60, n (%) |  |  |  | 0.637^1^ |  |  |  | 0.487^1^ |
| No | 13 (65.0) | 97 (58.4) | 110 (59.1) |  | 11 (45.8) | 43 (55.1) | 54 (52.9) |  |
| Yes | 7 (35.0) | 69 (41.6) | 76 (40.9) |  | 13 (54.2) | 35 (44.9) | 48 (47.1) |  |
| Missing | 8 | 130 | 138 |  | 41 | 39 | 80 |  |
| Worst grade of liver function-related adverse events at Day 60, n (%) |  |  |  | 0.632^2^ |  |  |  | 0.274^2^ |
| 0 | 13 (65.0) | 97 (58.4) | 110 (59.1) |  | 11 (45.8) | 43 (55.1) | 54 (52.9) |  |
| 1 | 5 (25.0) | 55 (33.1) | 60 (32.3) |  | 6 (25.0) | 23 (29.5) | 29 (28.4) |  |
| 2 | 2 (10.0) | 8 (4.8) | 10 (5.4) |  | 5 (20.8) | 7 (9.0) | 12 (11.8) |  |
| 3 | 0 (0.0) | 6 (3.6) | 6 (3.2) |  | 2 (8.3) | 5 (6.4) | 7 (6.9) |  |
| Missing | 8 | 130 | 138 |  | 41 | 39 | 80 |  |
| Stratification of the worst grade of liver function-related adverse events at Day 60, n (%) |  |  |  | 0.506^2^ |  |  |  | 0.426^2^ |
| 0 | 13 (65.0) | 97 (58.4) | 110 (59.1) |  | 11 (45.8) | 43 (55.1) | 54 (52.9) |  |
| 1-2 | 7 (35.0) | 63 (38.0) | 70 (37.6) |  | 11 (45.8) | 30 (38.5) | 41 (40.2) |  |
| 3 | 0 (0.0) | 6 (3.6) | 6 (3.2) |  | 2 (8.3) | 5 (6.4) | 7 (6.9) |  |
| Missing | 8 | 130 | 138 |  | 41 | 39 | 80 |  |
| Occurrence of grade ≥3 liver function-related adverse events at Day 60, n (%) |  |  |  | >0.999^1^ |  |  |  | 0.666^1^ |
| No | 20 (100.0) | 160 (96.4) | 180 (96.8) |  | 22 (91.7) | 73 (93.6) | 95 (93.1) |  |
| Yes | 0 (0.0) | 6 (3.6) | 6 (3.2) |  | 2 (8.3) | 5 (6.4) | 7 (6.9) |  |
| Missing | 8 | 130 | 138 |  | 41 | 39 | 80 |  |
| Occurrence of grade ≥2 liver function-related adverse events at Day 60, n (%) |  |  |  | 0.684^1^ |  |  |  | 0.143^1^ |
| No | 18 (90.0) | 152 (91.6) | 170 (91.4) |  | 17 (70.8) | 66 (84.6) | 83 (81.4) |  |
| Yes | 2 (10.0) | 14 (8.4) | 16 (8.6) |  | 7 (29.2) | 12 (15.4) | 19 (18.6) |  |
| Missing | 8 | 130 | 138 |  | 41 | 39 | 80 |  |
| Data are presented as n (%), percentages are based on non-missing value.  ^1^Fisher's exact test. ^2^Kruskal-Wallis rank sum test | | | | | | | | |

# Supplementary Table 9. Comparison of liver function-related adverse events among groups stratified by disease type

|  | **MgIG group** | | | | | **Control Group** | | | | |
| --- | --- | --- | --- | --- | --- | --- | --- | --- | --- | --- |
| **Characteristic** | **Lymphoma  N = 110** | **Myeloma  N = 93** | **Leukemia  N = 121** | **Overall (N = 324)** | **p-value** | **Lymphoma  N = 71** | **Myeloma  N = 46** | **Leukemia  N = 65** | **Overall (N = 182)** | **p-value** |
| Occurrence of liver function-related adverse events, n (%) |  |  |  |  | 0.593^1^ |  |  |  |  | 0.160^1^ |
| No | 52 (50.5) | 43 (48.9) | 64 (55.7) | 159 (52.0) |  | 26 (38.2) | 17 (38.6) | 15 (24.2) | 58 (33.3) |  |
| Yes | 51 (49.5) | 45 (51.1) | 51 (44.3) | 147 (48.0) |  | 42 (61.8) | 27 (61.4) | 47 (75.8) | 116 (66.7) |  |
| Missing | 7 | 5 | 6 | 18 |  | 3 | 2 | 3 | 8 |  |
| Worst grade of liver function-related adverse events, n (%) |  |  |  |  | 0.879^2^ |  |  |  |  | 0.059^2^ |
| 0 | 52 (50.5) | 43 (48.9) | 64 (55.7) | 159 (52.0) |  | 26 (38.2) | 17 (38.6) | 15 (24.2) | 58 (33.3) |  |
| 1 | 39 (37.9) | 32 (36.4) | 29 (25.2) | 100 (32.7) |  | 32 (47.1) | 20 (45.5) | 29 (46.8) | 81 (46.6) |  |
| 2 | 9 (8.7) | 9 (10.2) | 15 (13.0) | 33 (10.8) |  | 7 (10.3) | 4 (9.1) | 13 (21.0) | 24 (13.8) |  |
| 3 | 3 (2.9) | 4 (4.5) | 7 (6.1) | 14 (4.6) |  | 2 (2.9) | 3 (6.8) | 5 (8.1) | 10 (5.7) |  |
| 4 |  |  |  |  |  | 1 (1.5) | 0 (0.0) | 0 (0.0) | 1 (0.6) |  |
| Missing | 7 | 5 | 6 | 18 |  | 3 | 2 | 3 | 8 |  |
| Stratification of the worst grade of liver function-related adverse events, n (%) |  |  |  |  | 0.721^2^ |  |  |  |  | 0.159^2^ |
| 0 | 52 (50.5) | 43 (48.9) | 64 (55.7) | 159 (52.0) |  | 26 (38.2) | 17 (38.6) | 15 (24.2) | 58 (33.3) |  |
| 1-2 | 48 (46.6) | 41 (46.6) | 44 (38.3) | 133 (43.5) |  | 39 (57.4) | 24 (54.5) | 42 (67.7) | 105 (60.3) |  |
| 3 | 3 (2.9) | 4 (4.5) | 7 (6.1) | 14 (4.6) |  | 3 (4.4) | 3 (6.8) | 5 (8.1) | 11 (6.3) |  |
| Missing | 7 | 5 | 6 | 18 |  | 3 | 2 | 3 | 8 |  |
| Occurrence of grade ≥2 liver function-related adverse events, n (%) |  |  |  |  | 0.324^1^ |  |  |  |  | 0.112^1^ |
| No | 91 (88.3) | 75 (85.2) | 93 (80.9) | 259 (84.6) |  | 58 (85.3) | 37 (84.1) | 44 (71.0) | 139 (79.9) |  |
| Yes | 12 (11.7) | 13 (14.8) | 22 (19.1) | 47 (15.4) |  | 10 (14.7) | 7 (15.9) | 18 (29.0) | 35 (20.1) |  |
| Missing | 7 | 5 | 6 | 18 |  | 3 | 2 | 3 | 8 |  |
| Occurrence of grade ≥3 liver function-related adverse events, n (%) |  |  |  |  | 0.579^1^ |  |  |  |  | 0.729^1^ |
| No | 100 (97.1) | 84 (95.5) | 108 (93.9) | 292 (95.4) |  | 65 (95.6) | 41 (93.2) | 57 (91.9) | 163 (93.7) |  |
| Yes | 3 (2.9) | 4 (4.5) | 7 (6.1) | 14 (4.6) |  | 3 (4.4) | 3 (6.8) | 5 (8.1) | 11 (6.3) |  |
| Missing | 7 | 5 | 6 | 18 |  | 3 | 2 | 3 | 8 |  |
| Occurrence of liver function-related adverse events at Day 21, n (%) |  |  |  |  | 0.986^1^ |  |  |  |  | 0.502^1^ |
| No | 51 (62.2) | 49 (63.6) | 56 (62.9) | 156 (62.9) |  | 24 (52.2) | 14 (43.8) | 23 (41.1) | 61 (45.5) |  |
| Yes | 31 (37.8) | 28 (36.4) | 33 (37.1) | 92 (37.1) |  | 22 (47.8) | 18 (56.3) | 33 (58.9) | 73 (54.5) |  |
| Missing | 28 | 16 | 32 | 76 |  | 25 | 14 | 9 | 48 |  |
| Worst grade of liver function-related adverse events at Day 21, n (%) |  |  |  |  | 0.904^2^ |  |  |  |  | 0.550^2^ |
| 0 | 51 (62.2) | 49 (63.6) | 56 (62.9) | 156 (62.9) |  | 24 (52.2) | 14 (43.8) | 23 (41.1) | 61 (45.5) |  |
| 1 | 22 (26.8) | 21 (27.3) | 18 (20.2) | 61 (24.6) |  | 18 (39.1) | 16 (50.0) | 27 (48.2) | 61 (45.5) |  |
| 2 | 6 (7.3) | 5 (6.5) | 11 (12.4) | 22 (8.9) |  | 4 (8.7) | 2 (6.3) | 5 (8.9) | 11 (8.2) |  |
| 3 | 3 (3.7) | 2 (2.6) | 4 (4.5) | 9 (3.6) |  | 0 (0.0) | 0 (0.0) | 1 (1.8) | 1 (0.7) |  |
| Missing | 28 | 16 | 32 | 76 |  | 25 | 14 | 9 | 48 |  |
| Stratification of the worst grade of liver function-related adverse events at Day 21, n (%) |  |  |  |  | 0.971^2^ |  |  |  |  | 0.479^2^ |
| 0 | 51 (62.2) | 49 (63.6) | 56 (62.9) | 156 (62.9) |  | 24 (52.2) | 14 (43.8) | 23 (41.1) | 61 (45.5) |  |
| 1-2 | 28 (34.1) | 26 (33.8) | 29 (32.6) | 83 (33.5) |  | 22 (47.8) | 18 (56.3) | 32 (57.1) | 72 (53.7) |  |
| 3 | 3 (3.7) | 2 (2.6) | 4 (4.5) | 9 (3.6) |  | 0 (0.0) | 0 (0.0) | 1 (1.8) | 1 (0.7) |  |
| Missing | 28 | 16 | 32 | 76 |  | 25 | 14 | 9 | 48 |  |
| Occurrence of grade ≥3 liver function-related adverse events at Day 21, n (%) |  |  |  |  | 0.913^1^ |  |  |  |  | >0.999^1^ |
| No | 79 (96.3) | 75 (97.4) | 85 (95.5) | 239 (96.4) |  | 46 (100.0) | 32 (100.0) | 55 (98.2) | 133 (99.3) |  |
| Yes | 3 (3.7) | 2 (2.6) | 4 (4.5) | 9 (3.6) |  | 0 (0.0) | 0 (0.0) | 1 (1.8) | 1 (0.7) |  |
| Missing | 28 | 16 | 32 | 76 |  | 25 | 14 | 9 | 48 |  |
| Occurrence of grade ≥2 liver function-related adverse events at Day 21, n (%) |  |  |  |  | 0.308^1^ |  |  |  |  | 0.927^1^ |
| No | 73 (89.0) | 70 (90.9) | 74 (83.1) | 217 (87.5) |  | 42 (91.3) | 30 (93.8) | 50 (89.3) | 122 (91.0) |  |
| Yes | 9 (11.0) | 7 (9.1) | 15 (16.9) | 31 (12.5) |  | 4 (8.7) | 2 (6.3) | 6 (10.7) | 12 (9.0) |  |
| Missing | 28 | 16 | 32 | 76 |  | 25 | 14 | 9 | 48 |  |
| Occurrence of liver function-related adverse events at Day 30, n (%) |  |  |  |  | 0.443^1^ |  |  |  |  | 0.518^1^ |
| No | 35 (71.4) | 27 (58.7) | 52 (66.7) | 114 (65.9) |  | 13 (43.3) | 14 (53.8) | 16 (39.0) | 43 (44.3) |  |
| Yes | 14 (28.6) | 19 (41.3) | 26 (33.3) | 59 (34.1) |  | 17 (56.7) | 12 (46.2) | 25 (61.0) | 54 (55.7) |  |
| Missing | 61 | 47 | 43 | 151 |  | 41 | 20 | 24 | 85 |  |
| Worst grade of liver function-related adverse events at Day 30, n (%) |  |  |  |  | 0.345^2^ |  |  |  |  | 0.507^2^ |
| 0 | 35 (71.4) | 27 (58.7) | 52 (66.7) | 114 (65.9) |  | 13 (43.3) | 14 (53.8) | 16 (39.0) | 43 (44.3) |  |
| 1 | 13 (26.5) | 14 (30.4) | 18 (23.1) | 45 (26.0) |  | 14 (46.7) | 9 (34.6) | 18 (43.9) | 41 (42.3) |  |
| 2 | 1 (2.0) | 5 (10.9) | 3 (3.8) | 9 (5.2) |  | 1 (3.3) | 1 (3.8) | 4 (9.8) | 6 (6.2) |  |
| 3 | 0 (0.0) | 0 (0.0) | 5 (6.4) | 5 (2.9) |  | 1 (3.3) | 2 (7.7) | 3 (7.3) | 6 (6.2) |  |
| 4 |  |  |  |  |  | 1 (3.3) | 0 (0.0) | 0 (0.0) | 1 (1.0) |  |
| Missing | 61 | 47 | 43 | 151 |  | 41 | 20 | 24 | 85 |  |
| Stratification of the worst grade of liver function-related adverse events at Day 30, n (%) |  |  |  |  | 0.450^2^ |  |  |  |  | 0.580^2^ |
| 0 | 35 (71.4) | 27 (58.7) | 52 (66.7) | 114 (65.9) |  | 13 (43.3) | 14 (53.8) | 16 (39.0) | 43 (44.3) |  |
| 1-2 | 14 (28.6) | 19 (41.3) | 21 (26.9) | 54 (31.2) |  | 15 (50.0) | 10 (38.5) | 22 (53.7) | 47 (48.5) |  |
| 3 | 0 (0.0) | 0 (0.0) | 5 (6.4) | 5 (2.9) |  | 2 (6.7) | 2 (7.7) | 3 (7.3) | 7 (7.2) |  |
| Missing | 61 | 47 | 43 | 151 |  | 41 | 20 | 24 | 85 |  |
| Occurrence of grade ≥3 liver function-related adverse events at Day 30, n (%) |  |  |  |  | 0.089^1^ |  |  |  |  | >0.999^1^ |
| No | 49 (100.0) | 46 (100.0) | 73 (93.6) | 168 (97.1) |  | 28 (93.3) | 24 (92.3) | 38 (92.7) | 90 (92.8) |  |
| Yes | 0 (0.0) | 0 (0.0) | 5 (6.4) | 5 (2.9) |  | 2 (6.7) | 2 (7.7) | 3 (7.3) | 7 (7.2) |  |
| Missing | 61 | 47 | 43 | 151 |  | 41 | 20 | 24 | 85 |  |
| Occurrence of grade ≥2 liver function-related adverse events at Day 30, n (%) |  |  |  |  | 0.178^1^ |  |  |  |  | 0.747^1^ |
| No | 48 (98.0) | 41 (89.1) | 70 (89.7) | 159 (91.9) |  | 27 (90.0) | 23 (88.5) | 34 (82.9) | 84 (86.6) |  |
| Yes | 1 (2.0) | 5 (10.9) | 8 (10.3) | 14 (8.1) |  | 3 (10.0) | 3 (11.5) | 7 (17.1) | 13 (13.4) |  |
| Missing | 61 | 47 | 43 | 151 |  | 41 | 20 | 24 | 85 |  |
| Occurrence of liver function-related adverse events at Day 60, n (%) |  |  |  |  | 0.416^1^ |  |  |  |  | 0.591^1^ |
| No | 42 (59.2) | 25 (52.1) | 43 (64.2) | 110 (59.1) |  | 24 (55.8) | 15 (57.7) | 15 (45.5) | 54 (52.9) |  |
| Yes | 29 (40.8) | 23 (47.9) | 24 (35.8) | 76 (40.9) |  | 19 (44.2) | 11 (42.3) | 18 (54.5) | 48 (47.1) |  |
| Missing | 39 | 45 | 54 | 138 |  | 28 | 20 | 32 | 80 |  |
| Worst grade of liver function-related adverse events at Day 60, n (%) |  |  |  |  | 0.442^2^ |  |  |  |  | 0.502^2^ |
| 0 | 42 (59.2) | 25 (52.1) | 43 (64.2) | 110 (59.1) |  | 24 (55.8) | 15 (57.7) | 15 (45.5) | 54 (52.9) |  |
| 1 | 27 (38.0) | 17 (35.4) | 16 (23.9) | 60 (32.3) |  | 13 (30.2) | 6 (23.1) | 10 (30.3) | 29 (28.4) |  |
| 2 | 2 (2.8) | 4 (8.3) | 4 (6.0) | 10 (5.4) |  | 5 (11.6) | 2 (7.7) | 5 (15.2) | 12 (11.8) |  |
| 3 | 0 (0.0) | 2 (4.2) | 4 (6.0) | 6 (3.2) |  | 1 (2.3) | 3 (11.5) | 3 (9.1) | 7 (6.9) |  |
| Missing | 39 | 45 | 54 | 138 |  | 28 | 20 | 32 | 80 |  |
| Stratification of the worst grade of liver function-related adverse events at Day 60, n (%) |  |  |  |  | 0.487^2^ |  |  |  |  | 0.542^2^ |
| 0 | 42 (59.2) | 25 (52.1) | 43 (64.2) | 110 (59.1) |  | 24 (55.8) | 15 (57.7) | 15 (45.5) | 54 (52.9) |  |
| 1-2 | 29 (40.8) | 21 (43.8) | 20 (29.9) | 70 (37.6) |  | 18 (41.9) | 8 (30.8) | 15 (45.5) | 41 (40.2) |  |
| 3 | 0 (0.0) | 2 (4.2) | 4 (6.0) | 6 (3.2) |  | 1 (2.3) | 3 (11.5) | 3 (9.1) | 7 (6.9) |  |
| Missing | 39 | 45 | 54 | 138 |  | 28 | 20 | 32 | 80 |  |
| Occurrence of grade ≥3 liver function-related adverse events at Day 60, n (%) |  |  |  |  | 0.092^1^ |  |  |  |  | 0.283^1^ |
| No | 71 (100.0) | 46 (95.8) | 63 (94.0) | 180 (96.8) |  | 42 (97.7) | 23 (88.5) | 30 (90.9) | 95 (93.1) |  |
| Yes | 0 (0.0) | 2 (4.2) | 4 (6.0) | 6 (3.2) |  | 1 (2.3) | 3 (11.5) | 3 (9.1) | 7 (6.9) |  |
| Missing | 39 | 45 | 54 | 138 |  | 28 | 20 | 32 | 80 |  |
| Occurrence of grade ≥2 liver function-related adverse events at Day 60, n (%) |  |  |  |  | 0.059^1^ |  |  |  |  | 0.556^1^ |
| No | 69 (97.2) | 42 (87.5) | 59 (88.1) | 170 (91.4) |  | 37 (86.0) | 21 (80.8) | 25 (75.8) | 83 (81.4) |  |
| Yes | 2 (2.8) | 6 (12.5) | 8 (11.9) | 16 (8.6) |  | 6 (14.0) | 5 (19.2) | 8 (24.2) | 19 (18.6) |  |
| Missing | 39 | 45 | 54 | 138 |  | 28 | 20 | 32 | 80 |  |
| Data are presented as n (%), percentages are based on non-missing value.  ^1^Fisher's exact test. ^2^Kruskal-Wallis rank sum test | | | | | | | | | | |
|  | | | | | | | | | | |

**Supplementary Table 10 Incidence of adverse events observed in the study**

| **Characteristic** | **MgIG group (N = 324)** | **Control group (N = 182)** | **p-value^1^** |
| --- | --- | --- | --- |
| AE, n (%) |  |  |  |
| baseline | 15 (4.6) | 1 (0.5) | 0.014 |
| d21 | 6 (1.9) | 0 (0.0) | 0.092 |
| d30 | 2 (0.6) | 0 (0.0) | 0.538 |
| d60 | 7 (2.2) | 0 (0.0) | 0.053 |
| AE classified by PT |  |  |  |
| Low blood K+, n (%) |  |  |  |
| baseline | 4 (1.2) | 0 (0.0) | 0.302 |
| d21 | 2 (0.6) | 0 (0.0) | 0.538 |
| d30 | 1 (0.3) | 0 (0.0) | ＞0.999 |
| d60 | 2 (0.6) | 0 (0.0) | 0.538 |
| Palpitations or fatigue, n (%) |  |  |  |
| baseline | 3 (0.9) | 1 (0.5) | ＞0.999 |
| d21 | 0 (0.0) | 0 (0.0) |  |
| d30 | 0 (0.0) | 0 (0.0) |  |
| d60 | 0 (0.0) | 0 (0.0) |  |
| Nausea, vomiting, diarrhea, n (%) |  |  |  |
| baseline | 4 (1.2) | 0 (0.0) | 0.302 |
| d21 | 3 (0.9) | 0 (0.0) | 0.556 |
| d30 | 1 (0.3) | 0 (0.0) | ＞0.999 |
| d60 | 4 (1.2) | 0 (0.0) | 0.302 |
| Itching, rash, n (%) |  |  |  |
| baseline | 1 (0.3) | 0 (0.0) | ＞0.999 |
| d21 | 1 (0.3) | 0 (0.0) | ＞0.999 |
| d30 | 0 (0.0) | 0 (0.0) |  |
| d60 | 1 (0.3) | 0 (0.0) | ＞0.999 |

Data are presented as n (%).

^1^Fisher’s exact test

Abbreviation: MgIG: magnesium isoglycyrrhizinate

**Supplementary Figure**

**
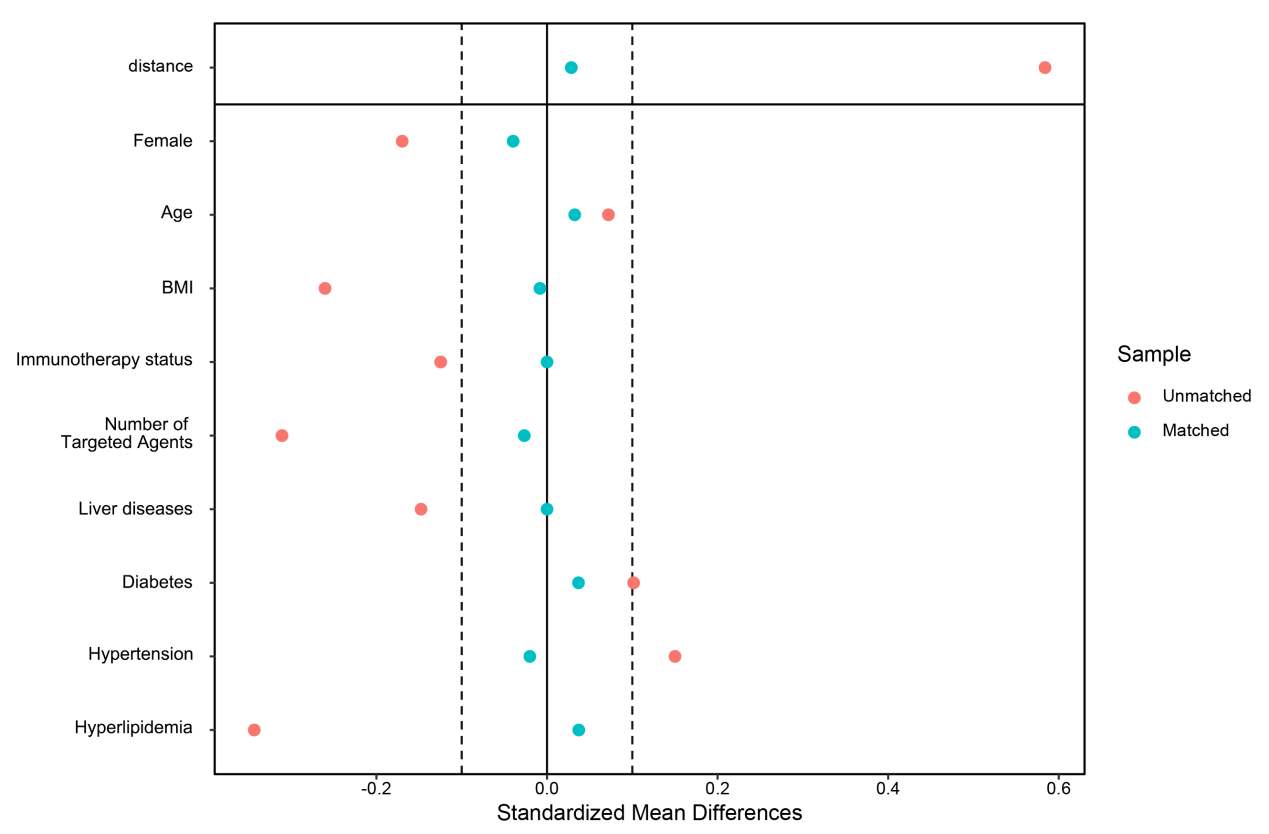
**

**Supplementary Figure 1** PSM results show balanced baseline characteristics for MgIG and control groups. Orange dots: pre-matching variables (most SMDs > 0.1); Green dots: post-matching variables (all SMDs < 0.1). SMD: standard mean differences.
